# An injectable, self-healing, anti-infective, and anti-inflammatory novel glycyrrhizic acid hydrogel for promoting acute wound healing and regeneration

**DOI:** 10.3389/fbioe.2024.1525644

**Published:** 2025-01-10

**Authors:** Qiyou Guo, Ruojing Li, Yeying Zhao, Huibo Wang, Wenqiang Luo, Junhao Zhang, Zhenlu Li, Peige Wang

**Affiliations:** ^1^ Department of Emergency Surgery, The Affiliated Hospital of Qingdao University, Qingdao, China; ^2^ Department of Emergency Medicine, Zhuji Affiliated Hospital of Wenzhou Medical University, Zhuji, Zhejiang, China

**Keywords:** glycyrrhizic acid, hydrogels, wound dressings, acute wounds, wound repair

## Abstract

**Introduction:**

Bacterial infection, a complex wound microenvironment, and a persistent inflammatory response in acute wounds can result in delayed healing and abnormal scar formation, thereby compromising the normal function and aesthetic appearance of skin tissue. This issue represents one of the most challenging problems in clinical practice. This study aims to develop a hydrogel dressing specifically designed for the treatment of acute wounds, providing immediate and effective protection for the affected areas. This innovation seeks to offer a novel and advanced solution for the management of acute wounds.

**Methods:**

In this study, a composite hydrogel scaffold was synthesized through the reaction between oxidized glycyrrhizic acid and carboxymethyl chitosan Schiff base. The material properties of the hydrogel were systematically characterized, and its biocompatibility and antibacterial efficacy were rigorously evaluated. A rat wound model was established to compare multiple groups, thereby assessing the impact of the hydrogel on the wound microenvironment and wound repair.

**Results:**

The results demonstrated that the OGA-CMCS hydrogel exhibited excellent injectability, biocompatibility, and antibacterial properties. It was capable of enhancing the wound microenvironment, which in turn influenced the polarization of macrophages from the M1 to the M2 phenotype, thereby mitigating the inflammatory response, promoting angiogenesis and granulation tissue regeneration, and accelerating wound healing.

**Discussion:**

This study successfully developed a novel glycyrrhizin-based hydrogel dressing, which not only introduces innovative approaches for the emergency management of acute surface wound defects but also provides an experimental foundation. It is anticipated to contribute significantly to addressing relevant clinical challenges.

## 1 Introduction

In the emergency treatment of multiple injuries, the attending physician must first address the issues of organ rupture, fractures, and massive hemorrhage; however, attention to some acute surface wounds is often seriously insufficient (Ubbink et al., 2015). The skin is a vital organ that envelops the body’s surface and has direct exposure to the external environment. When extensive areas of the skin are impaired or wounds remain unhealed over time, local and even systemic issues may occur, such as bacterial infections, metabolic disorders, and loss of immune function (Mas-Celis et al., 2021; Ma et al., 2016). Conventional wound dressings, such as gauzes and hemostatic bandages, tend to adhere to the wound and must be removed during multiple dressing changes, which can damage the regenerating skin tissue and increase the risk of secondary injury and infection (Ding et al., 2024; Muthuramalingam et al., 2019). Consequently, effectively preventing and managing infections poses a significant challenge in the treatment of acute wounds.

Hydrogels are polymer materials with a three-dimensional network structure, formed by the physical or covalent crosslinking of polymer chains (Li et al., 2022; Zhao et al., 2023). Due to the presence of numerous hydrophilic groups, hydrogels can absorb water several tens or even hundreds of times their own weight, exhibiting high hydrophilicity, water retention, and swelling properties (Gong et al., 2023; Chen et al., 2019; Liu et al., 2023). Additionally, hydrogels can cool wounds and reduce local temperatures associated with tissue inflammation reactions. Furthermore, hydrogels possess excellent biocompatibility and cell adhesion properties, allowing direct contact with damaged areas on the body’s surface to minimize fluid loss and prevent secondary infections from occurring (He et al., 2023; Chen et al., 2024; Yu et al., 2024).

Injectable hydrogels possess numerous significant characteristics and advantages. They exhibit excellent biocompatibility and are predominantly composed of natural polysaccharides (e.g., hyaluronic acid, alginate), proteins (e.g., collagen, gelatin), or modified synthetic polymers (Lan et al., 2024; Hernández-González et al., 2020). These materials demonstrate favorable interactions with human tissues and cells, thereby minimizing the risk of inflammation and immune rejection. For instance, injectable hydrogels derived from hyaluronic acid can establish a microenvironment that closely mimics natural tissue, facilitating cell adhesion, proliferation, and differentiation (Perera et al., 2024).

With injectable properties enabling minimally invasive drug delivery, hydrogels can be administered to target sites, such as lesions or injuries, using smaller needles or catheters. This approach reduces both surgical trauma and patient discomfort compared to traditional implantable materials (Kenchegowda et al., 2023; Hernández-González et al., 2020). Owing to its fluid properties, this material can efficiently fill a wide range of irregularly shaped tissue defects, conforming to the contours of the wound and providing consistent support and protection during the wound healing process and in tissue engineering applications. For instance, it can serve as a drug delivery vehicle or a cellular scaffold to facilitate the regeneration of bone tissue in the context of bone defects repair (Koh et al., 2020; Kalairaj et al., 2024).

The physical and chemical properties of the material, including gelation time, mechanical strength, and swelling, can be tailored through adjustments in composition, concentration, or crosslinking methods to fulfill diverse requirements. For instance, rapid gelation is suitable for hemostasis, while enhanced cross-linking is beneficial for long-term applications, thereby improving stability (Xiang et al., 2024). Additionally, the material possesses a drug slow-release capability, allowing drugs to be incorporated via physical embedding or chemical bonding, and gradually released within the body (Yang et al., 2024; El-Husseiny et al., 2024). This process prolongs the therapeutic effect, enhances bioavailability, and mitigates toxic side effects. An example of this is the localized and sustained release of antibacterial, which can prevent infections and reduce the risk of systemic drug resistance (Li et al., 2024).

The natural amphiphilic compound glycyrrhizic acid, extracted from licorice roots, possesses potent pharmacological activities, including antioxidant, anti-inflammatory, and immune-regulating effects. It can serve as a green biomaterial for the direct construction of gel network structures based on its own matrix (Qian et al., 2022; Sun et al., 2023; Zhang et al., 2024; Li et al., 2023). However, GA hydrogels formed solely through self-assembly exhibit inadequate mechanical properties and low stability (Fischer and Lutz-Bueno, 2024). Therefore, it is necessary to chemically crosslink them with other substances to enhance the internal structural stability of the hydrogel system (Sun et al., 2023).

Compared to chitosan, carboxymethyl chitosan (CMCS) exhibits significantly improved water solubility, adsorption, and moisturizing ability while retaining the strong biocompatibility, antibacterial activity, and hemostatic properties of chitosan (Do et al., 2022; Upadhyaya et al., 2014; Upadhyaya et al., 2013). CMCS is a cationic, water-soluble polysaccharide with active amino groups that can react with hydroxyl and aldehyde groups (Geng et al., 2023; Shang et al., 2024; Wu et al., 2024). Therefore, CMCS is easily modified to produce novel hydrogels. Hydrogels based on CMCS can reduce inflammatory cell infiltration, effectively remove reactive oxygen species, promote epithelialization and collagen deposition, thereby accelerating wound healing (Cai et al., 2024).

**SCHEME 1:**
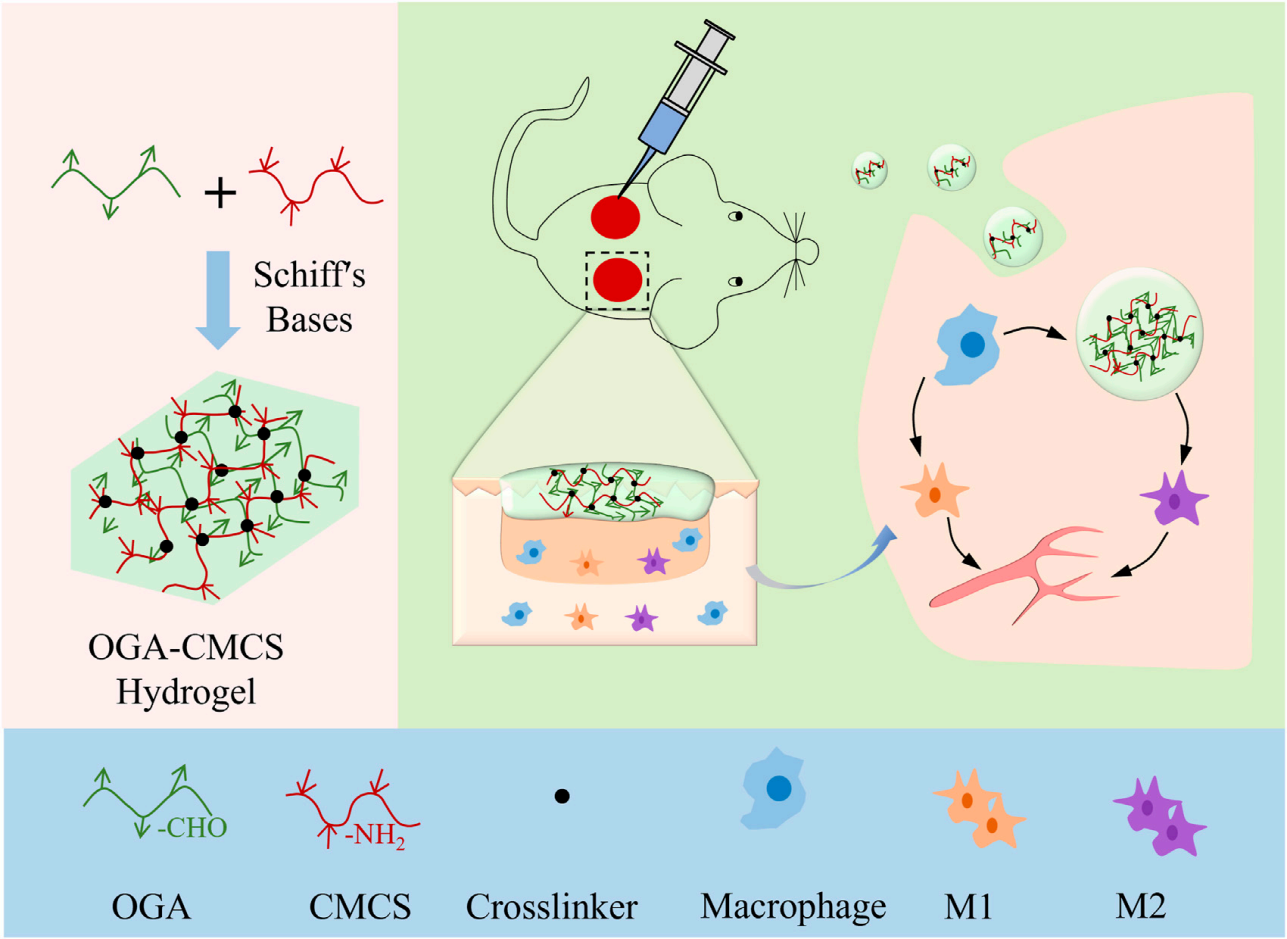
Schematic showing the fabrication of OGA/CMCS hydrogel and its potential application in wound healing.

Consequently, we synthesized a multifunctional hydrogel by integrating oxidized glycyrrhizic acid (OGA) and carboxymethyl chitosan (CMCS) through a dynamic Schiff base reaction. The hydrogel’s injectability, cytotoxicity, hemolysis, antibacterial activity, and anti-inflammatory properties were systematically evaluated. Ultimately, the hemostatic and wound healing efficacy of the hydrogel was assessed using mouse bleeding models and full-thickness skin defect models, respectively. The subsequent experimental results demonstrate that this multifunctional hydrogel exhibits notable antibacterial properties, promotes angiogenesis, and facilitates wound healing and tissue regeneration (Scheme 1).

## 2 Materials and methods

### 2.1 Materials

Glycyrrhizin is purchased from Meilunbio (Dalian, China). CMCS (American Chemical Society, ACS, ≥99.8%), sodium periodate (ACS, ≥99.8%), and sodium hydroxide solution (15%, w/v) were purchased from Aladdin (Shanghai, China). Hydrochloric acid (36%–38% wt%) was purchased from Jiuyi Reagent Co., LTD. (Shanghai, China). Methyl orange was purchased from Maclin (Shanghai, China). The compound colorant (sky blue and treasure red) was purchased from Shandong Dekai Food Co., LTD. (Shanghai, China). LB Nutrition AGAR Plate (9 cm) was purchased from Haibo Biotechnology Co., LTD., Qingdao High-tech Industrial Park (Qingdao, China).

### 2.2 Fabrication of hydrogel networks

#### 2.2.1 Synthesis of OGA

Based on the predetermined molar ratio (i.e., the mass of GA relative to its molecular weight to the mass of NaIO_4_ relative to its molecular weight), the required amount of NaIO_4_ was calculated and accurately weighed. Subsequently, NaIO_4_ was added to the GA solution and stirred vigorously in a dark environment for 5.5 h to ensure complete oxidation. Upon completion of the reaction, 2 mL of ethylene glycol was introduced, and stirring continued in the dark for an additional 0.5 h to effectively terminate the reaction. The obtained solution was collected, and the previously prepared dialysis membrane (molecular weight cutoff, 8–14 kDa) was used for dialysis for 72 h, changing the dialysis water every 8 h. Finally, the dialyzed solution was freeze-dried in a freeze-dryer. The powdered sample was collected after continuous freeze-drying for 36 h and sealed at 4°C for future use.

#### 2.2.2 Synthesis of OGA-CMCS hydrogel

The above synthesized OGA was dissolved in phosphate-buffered saline (PBS) at concentrations of 2%, 4%, or 6% (w/v). CMCS was dissolved in PBS at concentrations of 2%, 4%, or 6% (w/v). To create various concentrations of the completed hydrogels, 0.5 mL of each of the completely dissolved OGA and CMCS solutions was combined in 5 mL vials and allowed to stand.

#### 2.2.3 Gelation time

The gelation time of the OGA/CMCS hydrogels was measured and recorded using the vial tilt test, following the hydrogel preparation procedure described above. Specifically, 2.5 mL vials were tilted to observe the absence of liquid flow and the formation of gel within them. Each group of OGA/CMCS hydrogels was tested in triplicate.

#### 2.2.4 Fourier-transform infrared (FTIR) spectrometry

An FTIR spectrophotometer was applied to investigate the FTIR spectra of CMCS, GA, OGA, and OGA-CMCS hydrogels using the Nicolet-IS5 Spotlight from Thermo Electron at room temperature in the wave range of 4,000–500 cm^−1^ and the KBr pellet technique. The powders of each polymer were ground and mixed with dry KBr to form a disc, and 32 scans at a resolution of 4 cm^−1^ were used to record the spectra.

#### 2.2.5 Morphology

The microstructures and internal morphologies of the hydrogels were characterized using scanning electron microscopy (SEM). Three groups of cylindrical hydrogel samples (diameter 10 mm, height 10 mm), with varying degrees of oxidation, were freeze-dried and subsequently immersed in liquid nitrogen to achieve complete freezing. The frozen samples were then rapidly cut, and the resulting fracture surfaces were oriented upward. The samples were coated with gold for 45 s using a sputter coater set to a current of 10 mA. The cross-sectional morphologies of the hydrogel samples from each group were then examined and imaged using SEM at an accelerating voltage of 10 kV.

#### 2.2.6 Rheological property

Rheology primarily investigates the deformation and flow characteristics of materials under the influence of stress, strain, temperature, and time. For hydrogels, a unique class of materials that exhibit properties intermediate between solids and liquids, their rheological attributes hold particular significance. Oscillatory frequency sweep experiments were used to assess stability; oscillatory time sweep experiments were used to measure gelation kinetics; oscillatory strain sweep experiments were used to determine the viscoelastic region and critical strain; and alternating step strain sweep experiments were used to evaluate the self-healing properties (Zhao et al., 2023).

The storage modulus (G′) and loss modulus (G″) of OGA-CMCS-1, OGA-CMCS-2, and OGA-CMCS-3 were measured on a rheometer (model MCR302; Anton Paar Co., Ltd., Austria) at 25°C, and the gap between the parallel plates of the rheometer was kept at 1 mm. The experimental parameters were as follows:

(1) In the oscillation frequency sweep experiment, the constant strain was 1% and the angular frequency range was 0.1–100 rad/s.
(2) In the oscillation time sweep experiment, the fixed strain was 1% and the frequency was 10 Hz.
(3) In the oscillatory strain sweep experiment, the fixed frequency was 10 Hz and the strain range was 0.1%–1,000%.
(4) In the alternating step strain sweep experiment, the frequency was 10 Hz and the strain switched from 1% to 800%, with each cycle lasting 100 s.

#### 2.2.7 Degradability

The cylindrical OGA-CMCS-1, OGA-CMCS-2, and OGA-CMCS-3 hydrogels were synthesized. The initial mass of each sample was recorded as Wd. The hydrogels were placed into 37°C PBS solution (Xie et al., 2022; Yang et al., 2022), trypsin was added (the enzyme concentration was 0.1 mg/mL), and the hydrogels were removed at regular intervals. After removal, they were freeze-dried, and the mass of the lyophilized hydrogels was measured (recorded as Wt). Finally, the mass remaining (MR) of the hydrogels was determined using the following formula:

$$\text{MR}=\left(\text{Wt}/\text{Wd}\right)\times 100\%$$

The MR curve was drawn according to the calculated results.

#### 2.2.8 Swelling ratio (SR)

The cylindrical OGA-CMCS-1, OGA-CMCS-2, and OGA-CMCS-3 hydrogels were synthesized according to the above procedure (diameter: 10 mm; height: 10 mm). The initial mass of the hydrogels was recorded as Wd. The hydrogels were subsequently placed at 25 °C in 10 mL of PBS buffer and then removed at each preset time point. The surface of the hydrogels was wiped off with filter paper, and the hydrogels were immediately weighed; the weight was recorded as Wt. This process was repeated until equilibrium was attained. Finally, the SR of the hydrogels at the present moment was determined using the following formula:

$$\text{SR}=\left(\text{Wt}-\text{Wd}\right)\;/\text{ Wd}\times 100\%$$

The SR curve was drawn according to the calculated results.

#### 2.2.9 Self-healing ability

Two identical OGA-CMCS hydrogels were prepared in a circular mold, with one of them being stained red using rhodamine B dye. Each hydrogel sheet was then divided into four equal parts and rearranged. Subsequently, the hydrogels were left at room temperature for a duration of 4 h to observe their self-repair capabilities. Throughout the observation period, a smartphone was utilized to capture and record images every hour.

### 2.3 Cytocompatibility study

#### 2.3.1 Live/dead cell staining kit

The OGA-CMCS-1, OGA-CMCS-2, and OGA-CMCS-3 hydrogels were incubated in 5 mL of PBS. To prepare the hydrogel extract, the hydrogels were immersed in the liquid for 24 h. The extract was then sterilized under a UV lamp for 30 min. Subsequently, all cell experiments were conducted under strict aseptic conditions. Following the resuscitation, secondary passage, and cell counting of L929 cells, 0.1 mL of a 1.0 × 10^5^ cells/mL suspension was inoculated into each well of a 24-well plate, with 1 mL of DMEM (containing 10% fetal bovine serum, 100 IU/mL penicillin, and 100 μg/mL streptomycin) added to each well. The samples were divided into four groups: the PBS control group, the OGA-CMCS-1 group, the OGA-CMCS-2 group, and the OGA-CMCS-3 group, with three wells per group. The hydrogel extracts, consisting of 100 µL of PBS and each of the three samples, were filtered through a 0.22 µm filter to remove any bacterial contamination before being added to the corresponding wells. The cultures were then incubated at 37 °C in a 5% CO_2_ atmosphere.

#### 2.3.2 Cell counting kit-8 (CCK-8)

In this study, the CCK-8 assay was employed to assess the cell proliferation and cytotoxicity of the hydrogel extract. For cell staining, the experimental groups were identical: PBS control group, OGA-CMCS-1 group, OGA-CMCS-2 group, and OGA-CMCS-3 group. The hydrogel extract was prepared and sterilized using the same method as that used in the live/dead cell staining experiments. The cell suspension of L929 mouse fibroblasts was uniformly distributed in a 96-well plate at a density of 3 × 10^3^ cells per well (100 µL of DMEM per well) and cultured in an incubator maintained at 37 °C with 5% CO_2_ for 24 h. Based on the experimental design, 100 µL of PBS and the hydrogel extracts from the three samples, which had been sterilized by filtration through a 0.22 µm filter, were added to the designated wells. Additionally, blank wells (containing only medium without cells or extract) and control wells (containing medium and cells but no extract) were established in the remaining positions of the 96-well plate, and the culture was continued. Following co-cultivation for 24 and 48 h, 10 µL of CCK-8 reagent was added to each well, followed by an additional 3-h incubation at 37 °C. Subsequently, the absorbance of each well at 450 nm was quantified using a microplate reader. All experiments were conducted in triplicate (Yu et al., 2024).

#### 2.3.3 Test of hemolysis

The hydrogel was incubated with fresh rat heart blood for 1 h, followed by centrifugation at a speed of 2,000 revolutions per minute for 5 min. The absorbance of the supernatant at a wavelength of 540 nm was measured to assess hemolysis. Water served as the positive control, while saline served as the negative control. The hemolysis ratio was calculated using the following formula: hemolysis ratio = [(Ae-An)/(Ap-An)] × 100%, where Ae represents the absorbance value of the experimental group, Ap represents the absorbance value of the positive control group, and An represents the absorbance value of the negative control group.

### 2.4 *In vitro* antibacterial activity

#### 2.4.1 ZOI test

The prepared cylindrical hydrogel samples (10 mm in diameter and 10 mm in height) were sterilized by UV irradiation for 30 min and then divided into three groups. Subsequently, the samples were sequentially placed onto a solid nutrient medium uniformly coated with a bacterial suspension and incubated at 37°C for 12 h. Finally, the size of the antibacterial ring was measured.

#### 2.4.2 Agar disc diffusion test

The three groups of hydrogels were immersed in bacterial suspensions with a concentration of approximately 10^6^ CFU/mL and co-incubated at 37°C for 12, 24, and 48 h. After each time point, the absorbance of bacteria at 600 nm was measured using a microplate reader after dilution. Each experiment was repeated five times.

### 2.5 *In vivo* wound healing and histological analysis of hydrogel

This study involved 24 male SD rats, aged 8 weeks and weighing between 190 and 240 g, which were randomly assigned to four groups: Control, Alginate Ag, CMCS, and OGA-CMCS hydrogel groups, with six rats in each group. The rats underwent fasting the night prior to the experiment. Anesthesia was administered via intraperitoneal injection of pentobarbital at a dosage of 40 mg/kg. Following anesthesia induction, the rats were immobilized; their dorsal surfaces were disinfected before a full-thickness skin defect measuring 10 mm in diameter was created at the specified site. Each group received treatment with its corresponding dressing. Wound photographs were systematically captured at regular intervals to document the healing process. Rats were euthanized in batches on postoperative days 7 and 14 for sample collection intended for HE staining, Masson staining, immunofluorescence analysis, and ELISA.

### 2.6 Statistical analysis

All experiments were repeated three times. Data were analyzed using GraphPad Prism 9.0 (GraphPad Software, San Diego, CA, United States of America). All data are shown as mean ± standard error of the mean. Unpaired Student's t-test (two-tailed) or one-way analysis of variance was used for comparisons between groups. *p* < 0.05 was considered statistically significant.

## 3 Results and discussion

### 3.1 Synthesis of OGA-CMCS hydrogels

**FIGURE 1:**
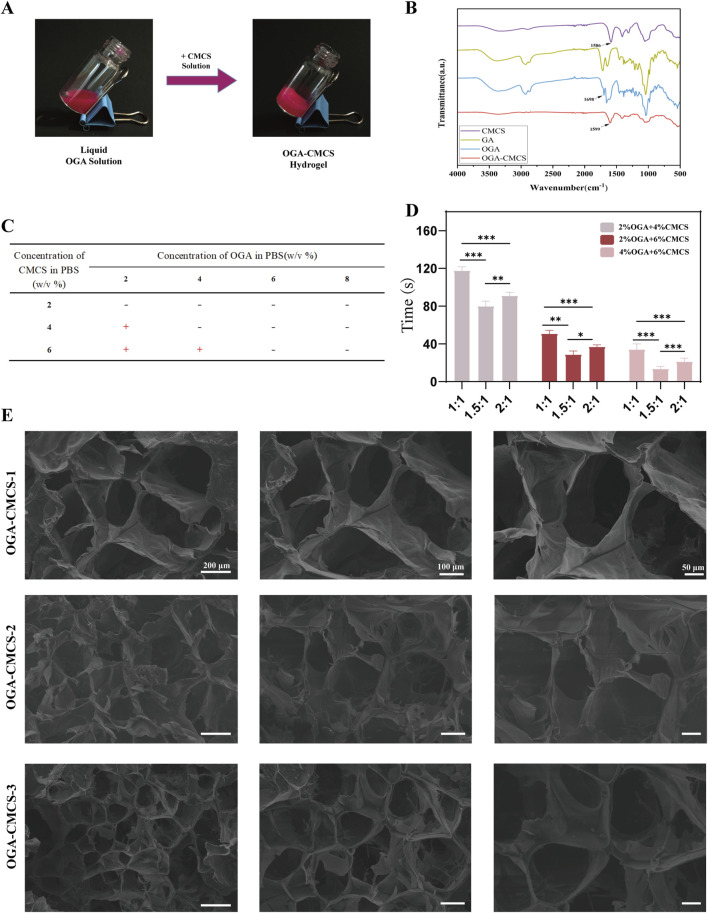
Synthesis of OGA-CMCS hydrogel. **(A)** Vial tilt test **(B)** Fourier transform infrared spectra of CMCS, OGA, GA, and OGA-CMCS hydrogels. **(C,D)** Gelation times of OGA-CMCS hydrogels with different mixing ratios. **(E)** Microstructures of OGA-CMCS-1, OGA-CMCS-2, and OGA-CMCS-3 hydrogels.

In addition, the gelation time was measured by a vial tilt test (Figure 1A). As shown in Figure 1C, the composites of 2% OGA + 4% CMCS, 2% OGA + 6% CMCS, and 4% OGA + 6% CMCS all formed gels within 3 min. The remaining groups were unable to reach the gel state even after being left for 24 h.

FTIR analysis was conducted on OGA, CMCS, GA, GA-CMCS, and OGA-CMCS. In the FTIR spectra, distinct absorption peaks corresponding to the characteristic functional groups of GA and CMCS were observed. Specifically, the amino group (-NH₂) in CMCS exhibited a characteristic absorption peak at 1,586 cm⁻^1^, while the carboxyl group (-COOH) in GA displayed its characteristic absorption peak at approximately 1,716 cm⁻^1^. Conversely, OGA demonstrated prominent absorption peaks for aldehyde and ketone groups at 1,698 cm⁻^1^, confirming the successful oxidation (Figure 1B).

GA-CMCS exhibited no characteristic peaks of aldehydes and ketones, nor the typical -C=N absorption peak (indicating the absence of Schiff base formation), but displayed a characteristic peak at 1632 cm⁻^1^ (Supplementary Figure S1A). This phenomenon can be attributed to the altered vibrational environment of the carbonyl group in the GA structure following its interaction with CMCS. When GA and CMCS are combined, the carboxyl groups of GA may form hydrogen bonds with the hydroxyl or amino groups of CMCS, leading to a shift in the vibrational frequency of the carbonyl group and a consequent peak shift. Additionally, physical adsorption or changes in molecular conformation also contribute to this effect. GA adsorption onto the CMCS molecular chain alters the conformation, thereby influencing the vibrational mode of the carbonyl group and resulting in the observed characteristic peak. Thus, it can be concluded that a successful Schiff base reaction occurs between the -CHO group present in OGA and the -NH_2_ group within CMCS (Figure 1B; Supplementary Figure S1A).

### 3.2 Gelation time

The hydrogel wound dressing should solidify into a gel-like consistency within tens of seconds of contact to create a moist healing environment. If the solidification time is too long, the wound remains exposed, increasing the risk of infection. If the solidification speed is too fast, it may not fully cover the wound, reducing adhesion and protective effects. The ideal dressing should solidify quickly, adhere to the wound, prevent bacterial invasion, and provide a moist environment for cell migration and proliferation (Peers et al., 2020).

As is shown in Figure 1D, the hydrogel formed from 4% OGA and 6% CMCS had the shortest gelation time, followed by the combination of 2% OGA and 4% CMCS. In contrast, the group with 2% OGA and 6% CMCS exhibited the longest gelation time (over 1 minute). Thus, a concentration ratio of 4% OGA to 6% CMCS appears optimal for gel formation. Notably, variations in GA oxidation degree also affect gelation time at equivalent concentrations. A molar ratio of NaIO_4_/GA at 1.5:1 resulted in shorter gelation times compared to a ratio of 2:1. Conversely, when this ratio is set to or below unity (1:1), longer gelation times were observed.

We prepared three groups of hydrogels with NaIO_4_/GA M ratios of 1:1, 1.5:1, and 2:1 at concentrations of 4% OGA and 6% CMCS, designated as OGA-CMCS-1, OGA-CMCS-2, and OGA-CMCS-3 for subsequent experiments.

### 3.3 DSC of the hydrogels

The crosslinking reaction represents a critical stage in the synthesis of hydrogels. Differential Scanning Calorimetry (DSC) serves as an effective tool for monitoring the thermal effects associated with crosslinking reactions, thereby facilitating the determination of kinetic parameters including the initial temperature, reaction rate, and extent of reaction. For instance, in the case of chemically crosslinked hydrogels, the exothermic peak observed during the crosslinking process via DSC provides valuable insights into the reaction dynamics. By analyzing the position, shape, and area of the exothermic peak, one can infer the progression of the crosslinking reaction, which in turn aids in optimizing the preparation conditions of the hydrogels, such as the concentration of the crosslinking agent, reaction temperature, and duration, ultimately leading to the production of hydrogels with desired characteristics (Castillo-Paz et al., 2024; Saletti et al., 2024).

Carboxymethyl chitosan (CMCS), a polysaccharide with a melting temperature (Tm) of 190°C, displays certain flexible and amorphous characteristics in its molecular chain. Conversely, glycyrrhizin (GA), an organic molecule characterized by a specific ring structure, has a melting temperature (Tm) of 184°C (Supplementary Figure S1B). These distinct thermal properties are attributed to the differences in molecular structure between the two materials, which can be further influenced by composite formation or chemical modifications.

The mechanism underlying the alteration in the crystallization temperature of oxidized glycyrrhizic acid (OGA) was investigated. It is hypothesized that the primary cause of this alteration is the substantial disruption of molecular symmetry and the equilibrium of intermolecular forces, induced by the functional groups generated during the oxidation process. Upon heating, the temperature associated with molecular reorganization and crystallization exhibits corresponding variations. Specifically, glycyrrhizic acid (GA) forms an aldehyde group following the oxidation reaction, and this transformation significantly modifies the original functional group composition of GA, thereby profoundly affecting the intermolecular interactions. Consequently, the cold crystallization temperature (Tcc = 243°C) and glass transition temperature (Tg = 369°C) of OGA demonstrate a pronounced change, which is closely linked to the aforementioned molecular structural modifications and aligns with established chemical principles and theories (Supplementary Figure S1B).

In the OGA-CMCS system, the amino (-NH_2_) groups of CMCS and the aldehyde (-CHO) groups of OGA undergo a Schiff base reaction. This interaction alters the mobility and arrangement of the molecular chains. Compared to CMCS alone, the presence of OGA in the OGA-CMCS system enhances intermolecular interactions, thereby restricting the mobility of CMCS molecular chain segments. As a result, the cold crystallization temperature (Tcc = 256°C) and the glass transition temperature (Tg = 389°C) are elevated (Supplementary Figure S1B). Additionally, this interaction disrupts the relatively ordered crystalline structure of OGA, leading to the disappearance or diminution of the melting point peak.

### 3.4 XPS of the hydrogels

XPS is a versatile technique that can be employed for elemental qualitative analysis to identify the types of elements present on the surface of a sample, quantitative analysis to determine the elemental content, chemical state analysis to elucidate the electronic configurations of elements, and depth profiling to obtain information on the elemental and chemical states at varying depths within the sample. This technique has significant applications in diverse fields, including materials science, chemistry, and semiconductor technology (Wang et al., 2025).

**FIGURE 2:**
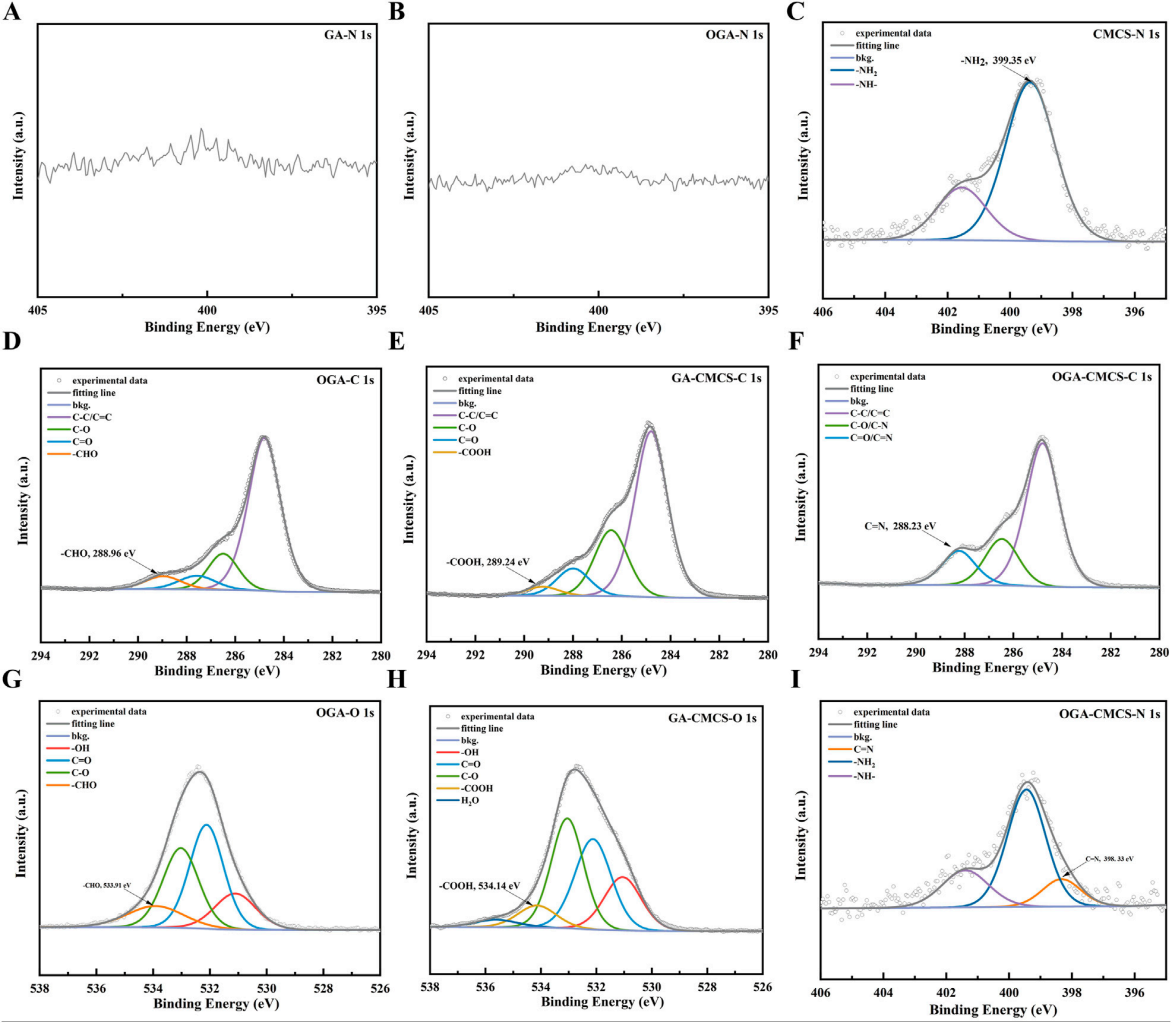
XPS of the hydrogels. **(A, B, C and I)** The N 1s fine spectrum of GA, OGA, CMCS, and OGA-CMCS. **(D, E and F)** The C 1s fine spectrum of OGA, GA-CMCS, and OGA-CMCS. **(G, H)** The O 1s fine spectrum of OGA and GA-CMCS.

Figures 2A, B show that the signal-to-noise ratio (SNR) of the N fine structure spectrum in the OGA and GA groups is very poor, and N is essentially absent. Figure 2C presents the N 1s fine spectrum data of CMCS, where the -NH₂ peak is identified with a binding energy of 399.35 eV. Among these, Figure 2D and G present the C 1s and O 1s fine spectrum of OGA, with binding energies of 288.96 eV and 533.91 eV, respectively. These findings indicate the presence of aldehyde (-CHO) peaks, thereby confirming the successful oxidation of GA.

Figures 2D, G present the C 1s and O 1s fine spectra of OGA, with binding energies of 288.96 eV and 533.91 eV, respectively, indicating the presence of aldehyde (-CHO) peaks. Figures 2E, F display the C 1s and O 1s fine spectra of GA-CMCS, with binding energies of 289.24 eV and 531.67 eV, respectively, suggesting the presence of carboxyl (-COOH) peaks. These results demonstrate that no new chemical bonds form between GA and CMCS upon simple mixing, which is consistent with the earlier conclusions from FTIR (Figure 1B; Supplementary Figure S1A) and DSC (Supplementary Figure S1B) analyses, further confirming the successful oxidation of GA to form aldehyde (-CHO). Figure 2F and I present the C 1s and N 1s fine spectrum of OGA-CMCS, The binding energies of 288.23 eV and 398.33 eV, respectively, for the aldehyde group of OGA and the amino group of CMCS, indicate that a Schiff base reaction has occurred between these functional groups, resulting in the formation of a new C=N bond. This finding is consistent with the previous FTIR (Figure 1B; Supplementary Figure S1A) and DSC (Supplementary Figure S1B) analyses results, further confirming the occurrence of the Schiff base reaction.

### 3.5 Morphology

The porous architecture of hydrogels promotes cellular proliferation and intercellular interactions (Wei et al., 2020; Xu et al., 2022; Yin et al., 2024). The microscopic morphology and pore structures of OGA-CMCS-1, OGA-CMCS-2, and OGA-CMCS-3 hydrogels are shown in Figure 1E. In this study, all three hydrogels demonstrated typical porous network structures characterized by uniform pore distribution and orderly arrangement.

The mean pore sizes of the OGA-CMCS-1, OGA-CMCS-2, and OGA-CMCS-3 hydrogels were 131.5 ± 22.25 μm, 76.58 ± 17.60 μm, and 92.06 ± 18.32 μm, respectively (Supplementary Figures S2A, B). As the concentration of CMCS increased, the hydrogel pore size initially increased and then decreased. Specifically, when OGA-CMCS-2 was utilized, the sponge porosity reached 95% (Supplementary Figure S2C). This phenomenon can be attributed to the fact that as the CMCS concentration rises, the distance between molecular chains diminishes, leading to increased molecular chain entanglement, higher density, and ultimately reduced porosity. Furthermore, no significant agglomerated particles were observed in any of the hydrogels, indicating that OGA and CMCS were effectively dispersed within the hydrogel system.

### 3.6 Rheological properties

**FIGURE 3:**
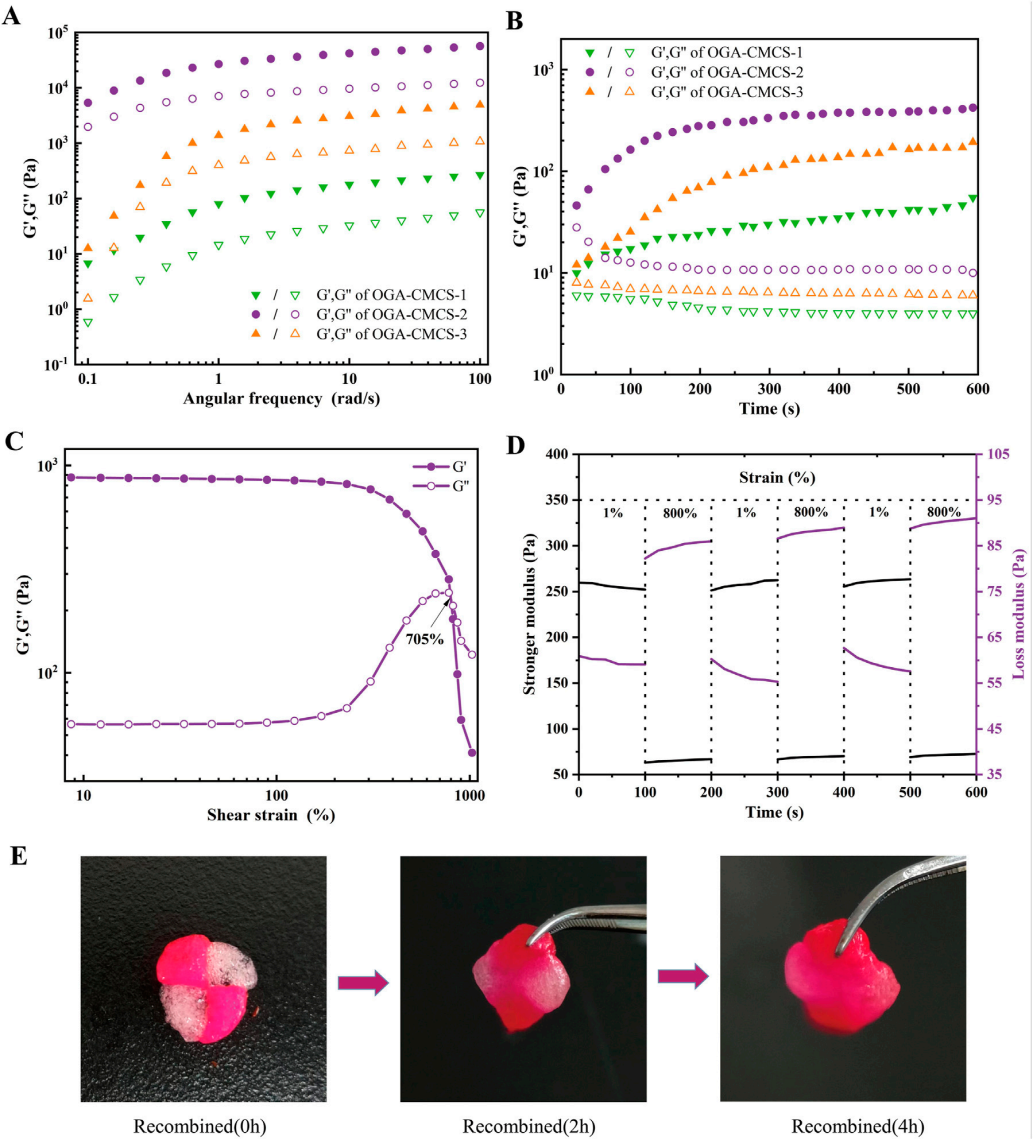
Results of the rheological property tests. **(A)** Oscillation frequency sweep experiments. **(B)** Oscillation time sweep experiments. **(C)** Oscillatory strain sweep experiments. **(D)** Alternating step strain sweep experiments. **(E)** Self-healing process of OGA-CMCS-2 hydrogel.

In the final curves obtained from both the oscillation frequency scanning experiment and the oscillation time scanning experiment (Figures 3A, B), it is observed that OGA-CMCS-1, OGA-CMCS-2, and OGA-CMCS-3 hydrogels consistently exhibit G′ exceeding G″ across the entire frequency or time spectrum. This indicates that the hydrogels consistently exhibit stable viscoelastic solid behavior throughout the experiments, remaining unaffected by significant morphological changes despite variations in frequency or time.

### 3.7 Self-healing of the hydrogels

Self-healing materials are a class of intelligent materials that possess the intrinsic capability to autonomously repair damage without external intervention (Tu et al., 2019). Due to daily physical activities, the implanted hydrogels will continuously be subjected to mechanical forces, which may lead to the destruction of the network structure’s integrity. The destruction of the hydrogel structure not only reduces its functional performance, but also increases the risk of infection due to microbial invasion into the cavity or cracks (Hafezi et al., 2021).

In the case of the OGA-CMCS-2 hydrogel, this study initially determined the maximum strain value that the hydrogel can withstand by analyzing the strain sweep rheological test curve of the sample. As illustrated in Figure 3C, with increasing strain values, the curves representing G′ and G″ intersect at γ = 705%. At this juncture, G′ is equal to G''. However, as the strain continues to increase beyond this point, G′ surpasses G″, indicating that the three-dimensional network of the hydrogel has been compromised, and the sample transitions from a gel state to a sol state. Consequently, it can be concluded that the maximum strain value tolerated by this hydrogel is 705%.

Subsequently, alternating stepped strain tests were performed on the hydrogels using a small strain of γ = 1% and a large strain of γ = 800%, as illustrated in Figures 3D, E. The results from the three rheological tests, which involved cycles of small and large strain applied under large strain conditions, reveal that G′ exceeds G″ during the application of the large strain, indicating that the hydrogel’s network structure is compromised and the hydrogel transitions to a sol state. Upon removal of the large stress, both G′ and G″ rapidly recover to their pre-damage modulus values under small strain conditions, demonstrating that the hydrogel possesses excellent self-healing capabilities.

### 3.8 Compression performance, adhesive properties, and injectability of the hydrogels

**FIGURE 4:**
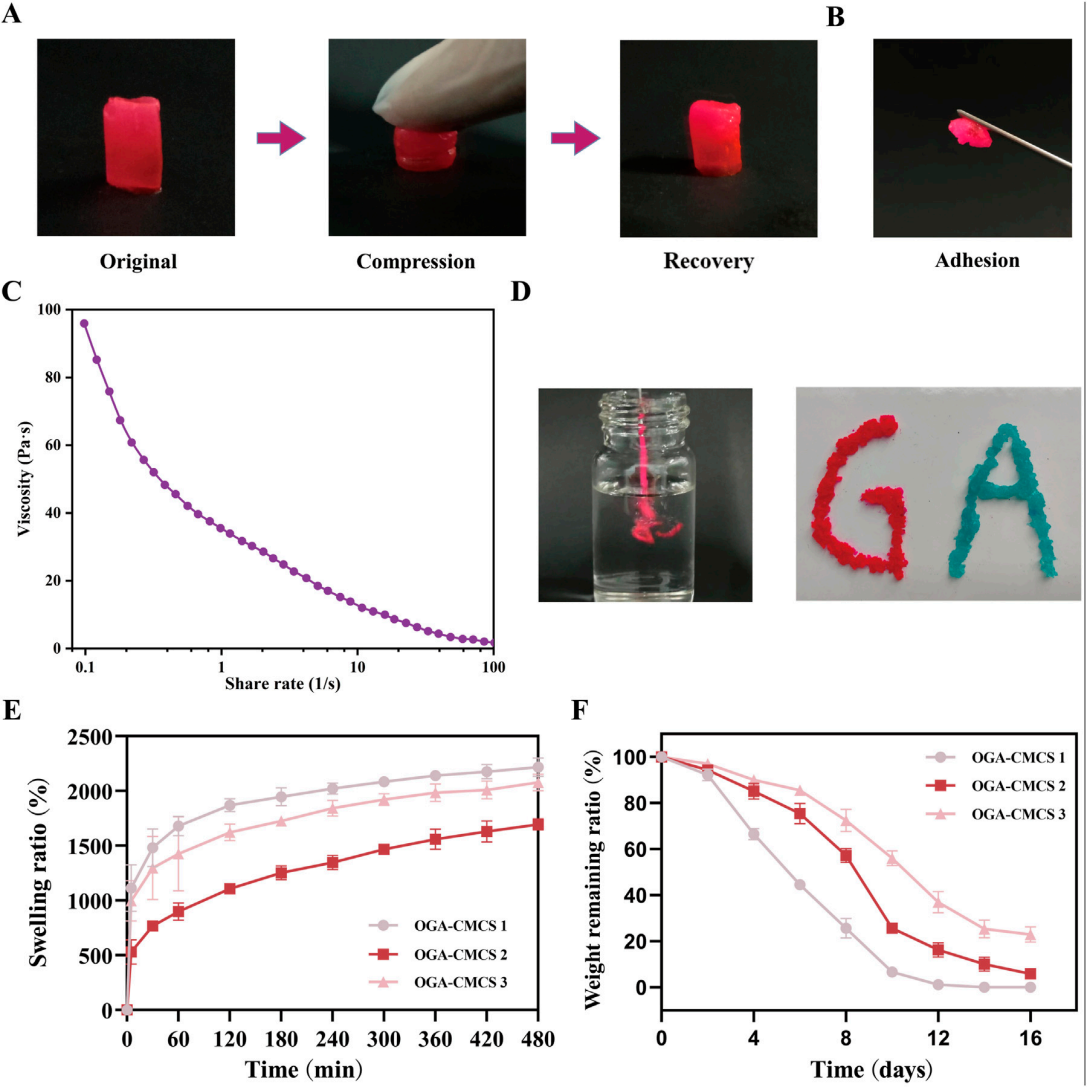
Materialistic characterization of OGA-CMCS hydrogel. **(A)** Realistic perspective of OGA-CMCS-2 hydrogel after compression and recovery. **(B)** Adhesion of OGA-CMCS-2 hydrogel. **(C)** The relationship between the viscosity of OGA-CMCS-2 hydrogel and shear rate. **(D)** Injectable properties of OGA-CMCS-2 hydrogel. **(E)** Swelling ratios of OGA-CMCS-1, OGA-CMCS-2, and OGA-CMCS-3 hydrogels. **(F)** The degradation rates of OGA-CMCS-1, OGA-CMCS-2, and OGA-CMCS-3 (Note: n = 4, *P* < 0.05, **P < 0.01, ***P < 0.001).

Figure 4A shows the realistic view of the OGA-CMCS-2 hydrogel undergoing compression and recovery under external force. Figure 4B illustrates that the hydrogel exhibits excellent adhesive properties, ensuring it remains securely attached when in contact with various objects.

Figure 4C demonstrates that an increase in shear rate results in a reduction of the hydrogel’s viscosity, thereby confirming its shear-thinning properties, which facilitate its injectable behavior. Furthermore, Figure 4D provides a more intuitive illustration showing that the hydrogel can flow seamlessly through the syringe needle and be extruded as ‘GA’ under shear-thinning conditions without causing any blockage of the needle.

### 3.9 Swelling ratio and degradation properties

Wounds have been documented to heal more efficiently in humid conditions; however, excessive moisture can be detrimental to the wound healing process due to an increased risk of infection and maceration (Wang et al., 2025). The water absorption capacity of hydrogel dressings is one of their fundamental properties. On the one hand, adequate liquid absorption is essential for controlling hemorrhage and absorbing exudate from injured areas. On the other hand, appropriate swelling characteristics help maintain moisture at the wound site, which is conducive to the formation of epithelial tissue (Jian et al., 2019).

The hydrogel exhibits enhanced absorbency of tissue exudates, while its inherent softness mitigates the detrimental effects of tissue friction (Park et al., 2022; Chellathurai et al., 2024). Following the immersion of three groups of freeze-dried hydrogels with varying degrees of oxidation in a humid environment created by PBS (Figure 4E), the swelling rates stabilized within approximately 8 h. Notably, the OGA-CMCS-1 hydrogel exhibited the highest swelling rate, reached 2218.48% ± 84.97%; this was followed by the OGA-CMCS-3 hydrogel, which demonstrated a swelling rate of 2077.94% ± 75.87%. In contrast, the OGA-CMCS-2 hydrogel displayed the lowest swelling rate of 1694.73% ± 50.90%.

All the hydrogels underwent complete degradation within 14 days after immersion in PBS (Figure 4F). As the degree of GA oxidation increased, there was a noticeable deceleration in the degradation rate of the hydrogel. This observation suggests that the degradation kinetics of the hydrogel are predominantly influenced by its cross-linking density.

The excellent swelling properties and biodegradability of hydrogels effectively maintain a moist environment at the wound site. This moisture retention facilitates cell migration, proliferation, and differentiation. Additionally, a moist environment prevents the formation of scabs, thereby reducing pain and minimizing the risk of scar formation associated with scabbing. As the hydrogel degrades, it interacts favorably with surrounding tissue cells, providing a gentle and conducive environment for wound healing (Jian et al., 2019; Li et al., 2022; Wang et al., 2025).

Based on these findings, OGA-CMCS-2 hydrogel was chosen for subsequent material characterization and animal experiments due to its superior tensile strength, adhesive properties, degradation rate, and release performance compared to OGA-CMCS hydrogels with different concentrations.

### 3.10 Cytocompatibility of hydrogels

**FIGURE 5:**
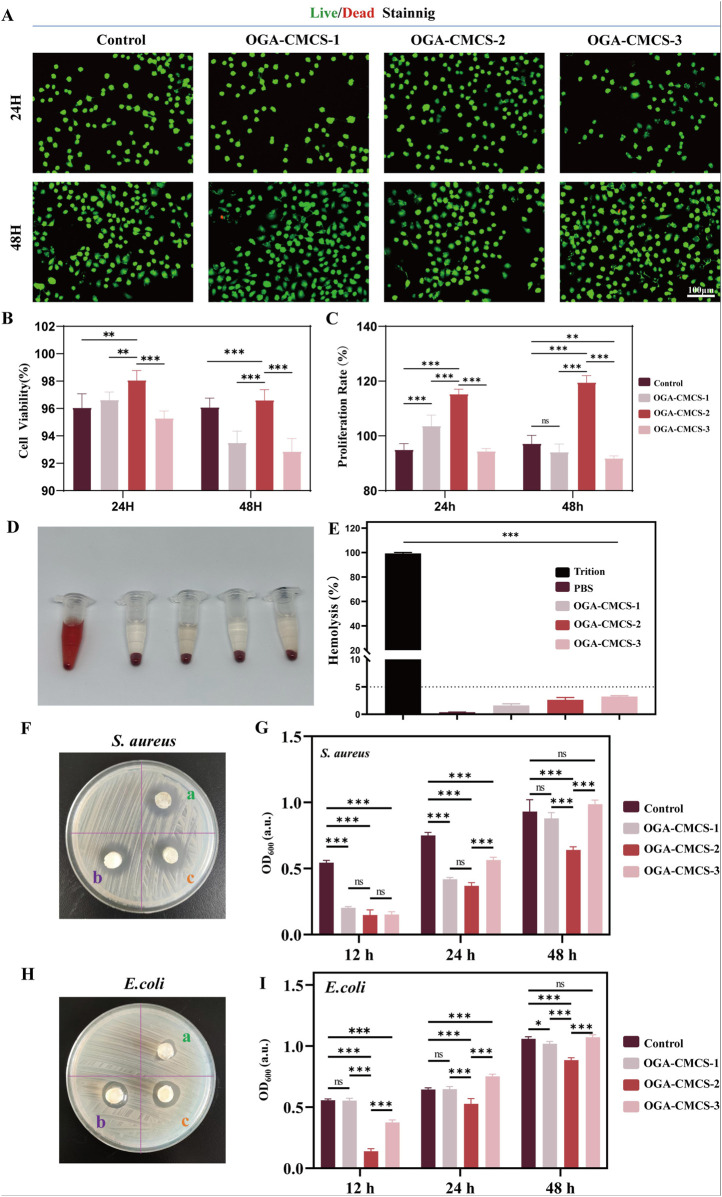
Biocompatibility and antibacterial activity of OGA-CMCS hydrogels. **(A)** Live/dead staining of fibroblasts after co-culture with hydrogel leachate. **(B)** Quantitative analysis of L929 fibroblast survival. **(C)** CCK-8 assay of fibroblast cell proliferation rate after 24 h and 48 h of culture in BMDM medium and leachate. **(D)** The hemolysis situation of red blood cells following different treatments. **(E)** The comparison of hemolysis rates following different treatments. **(F, H)** ZOI test and Agar disc diffusion test were used to evaluate the antibacterial activity of OGA-CMCS hydrogels against *Staphylococcus aureus* and *Escherichia coli*. **(G, I)** Optical density (OD) was used to compare the bacterial density between groups. (Note: n = 4, *P* < 0.05, **P < 0.01, ***P < 0.001).

Good cytocompatibility is essential for materials used in biomedical applications (Song et al., 2024; Liu et al., 2022). This study evaluated the biocompatibility of OGA-CMCS hydrogels at the cellular level. Live/dead cell staining experiments were performed, and the results shown in Figure 5A indicate that only a small number of dead cells were observed in OGA-CMCS-1 hydrogels and OGA-CMCS-3 hydrogels even after 24 and 48 h of co-culture with L929 mouse fibroblasts. Quantitative analysis showed that the survival rate of fibroblasts exceeded 90% (Figure 5C), and there was no significant difference between the experimental group and the control group. We then performed further hemolysis experiments. In the hemolysis test (Figure 5D), the supernatant of the positive control group turned red due to the lysis of red blood cells and the release of their contents, indicating severe hemolysis. In contrast, the supernatant of the hydrogel group remained optically clear, indicating the integrity of the red blood cells, which suggests that the OGA-CMCS hydrogel has good blood compatibility. In addition, the results showed that the hemolysis rate of all hydrogel groups was less than 5% (Figure 5E), which confirmed the lack of cytotoxicity and good biocompatibility of the three hydrogels.

### 3.11 Antibacterial properties

The antibacterial properties of hydrogels are of significant importance. In wound care, they can effectively prevent bacterial infections, foster an optimal environment for wound healing, and minimize the occurrence of complications. Regarding medical implant materials, antibacterial hydrogels can mitigate the risk of post-implantation infections and extend the longevity of the materials (Xue et al., 2019; Thapa et al., 2020).

The antibacterial activity is essential for the management of infected wounds, with *Escherichia coli* (a prototypical Gram-negative bacterium) and *S. aureus* (a prototypical Gram-positive bacterium) being prevalent pathogens responsible for wound infections (Zhao et al., 2020; Wu et al., 2024). Figures 5F, H show that the hydrogels have antibacterial effects against both bacterial strains. With PBS buffer as a control, Figure 5G and I show the OD600 values of the bacterial suspensions incubated with the three hydrogels for 12 h, 24 h, and 48 h. The smaller the OD600 value, the better the antibacterial effect. The results show that all three hydrogels exhibit antibacterial activity against *S. aureus* after 12 h of co-incubation, while the OGA-CMCS-2 hydrogel exhibits better antibacterial activity than the other two at 24 h and 48 h. For *E. coli*, the OGA-CMCS-2 hydrogel shows the strongest antibacterial activity at all three time points. The OGA-CMCS-1 hydrogel has a significantly higher OD600 value than the other two at 12 h of co-incubation, indicating that its antibacterial activity against *E. coli* is weaker than that of the other hydrogels.

Considering the comprehensive experimental results, the OGA-CMCS-2 hydrogel demonstrated superior performance compared to the other groups in terms of material characterization, biocompatibility, and *in vitro* antibacterial efficacy. Consequently, we have chosen the OGA-CMCS-2 hydrogel for subsequent animal studies.

### 3.12 *In vivo* wound healing and histological analysis of hydrogel

#### 3.12.1 Analysis of closure rate of full-thickness skin defects in rats

**FIGURE 6:**
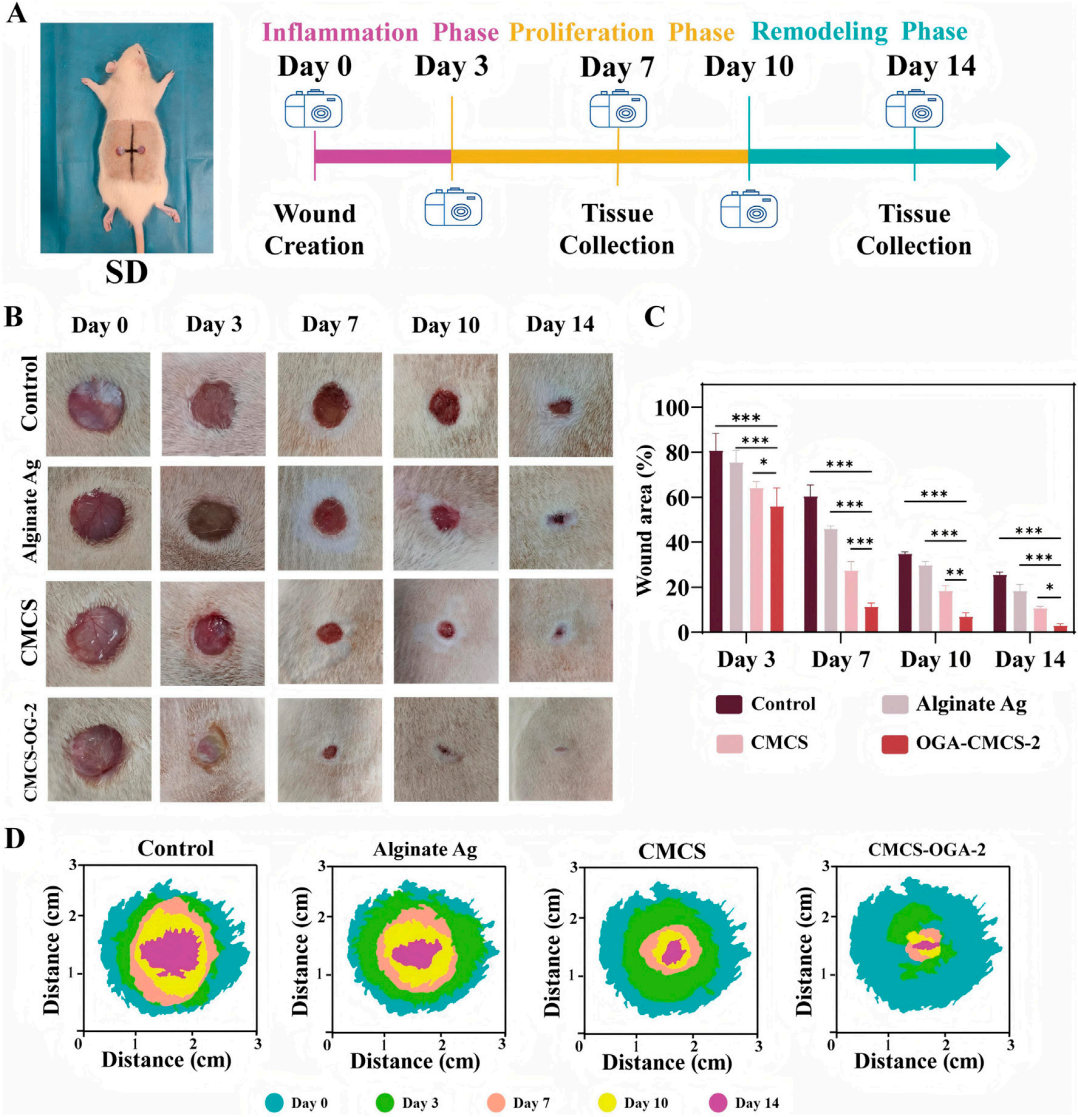
Evaluation of the Efficacy of Hydrogel in Acute Wound Healing **(A)** Schematic representation of experimental animal modeling and timeline; **(B)** Representative images of wound sites at designated time points for each treatment group; **(C)** Statistical analysis of wound area across the different treatment groups. **(D)** Schematic representation of the wound healing process. (Note: n = 4, *P* < 0.05, **P < 0.01, ***P < 0.001).

We assessed the efficacy of OGA-CMCS-2 hydrogel in promoting wound healing through a series of *in vivo* experiments. The experimental protocol for the animal study is illustrated in Figure 6A.

In the full-thickness skin defect model of SD rats, the OGA-CMCS-2 hydrogel dressing group significantly enhanced the healing rate of acute defects compared to the blank control group, Alginate Ag group, and CMCS group.

From the data presented in Figures 6B, D, it is evident that wounds treated with OGA-CMCS hydrogel dressings exhibited significant advantages. On the 7th day post-surgery, the wound closure rate was notably faster compared to the other three groups, indicating the unique efficacy of this hydrogel dressing in promoting early wound healing. This enhanced healing may be attributed to the specific structure and composition of the OGA-CMCS hydrogel, which effectively regulates the local wound microenvironment by maintaining optimal moisture levels, facilitating the release and retention of cytokines, and thus accelerating cell migration, proliferation, and extracellular matrix synthesis—key processes in wound healing.

Complete wound closure was observed by day 10, further validating the sustained effectiveness of the OGA-CMCS hydrogel dressing throughout the entire wound healing process. This sustained effectiveness can likely be attributed to its excellent biocompatibility, which supports continuous tissue repair and regeneration in a mild and favorable environment, while simultaneously reducing hindering factors such as excessive inflammatory responses and the risk of infection.

Furthermore, as depicted in Figure 6C, the closure rate for the OGA-CMCS hydrogel dressing group reached 88.59% ± 1.58% on postoperative day 7 and increased to 97.00% ± 0.80% by postoperative day 14. In contrast, the closure rates for the other three groups on postoperative day 14 were recorded at 74.36% ± 1.07%, 81.63% ± 2.90%, and 89.25% ± 0.77%, respectively.

#### 3.12.2 Histological analysis of hydrogels

The reduction of inflammatory cells is crucial for effective wound healing. Initially, the inflammatory response facilitates the removal of necrotic tissue and pathogens through the aggregation of neutrophils and macrophages (Liu et al., 2023; Yin et al., 2024; Zhao et al., 2020). However, prolonged elevated levels of inflammation can result in chronic inflammation, leading to the excessive release of inflammatory mediators, inhibition of fibroblast activity, impairment of extracellular matrix synthesis and deposition, disruption of neovascularization, and overall hindrance to the wound healing process. Consequently, the decline in inflammatory cell count signifies that inflammation is being effectively managed, promoting the transition of the wound from the inflammatory phase to the proliferative phase, and establishing optimal conditions for fibroblast proliferation, migration, and neovascularization (Cai et al., 2024; Song et al., 2024; Xue et al., 2019; Wang et al., 2025).

The proliferation of fibroblasts is critical for the wound healing process. As one of the primary reparative cells, fibroblasts play a pivotal role in this phase (Cariba et al., 2024). During the proliferative stage, the augmented population of fibroblasts actively synthesizes and secretes a substantial amount of extracellular matrix components, including collagen and elastin fibers. Collagen is integral to the development of granulation tissue, providing structural support to the wound site. This process facilitates wound contraction, fills the tissue defect, and promotes the repair and remodeling of the wound. Additionally, it enhances the mechanical properties of the wound, contributing to the restoration of normal skin function and appearance (Hao et al., 2024; Naghib et al., 2024).

Enhanced neovascularization is essential for wound repair. This process facilitates the efficient transport of nutrients and metabolic waste, serving as a vital conduit. Neovascularization delivers oxygen, amino acids, glucose, and other essential nutrients to the wound site, which is crucial for the survival and function of fibroblasts. Additionally, it removes waste products such as carbon dioxide and lactic acid, ensuring normal cellular metabolism. The progressive development of the neovascular network at the wound site accelerates healing, enhances repair quality, and reduces the risk of infection, non-healing, or scar formation (Cariba et al., 2024; Hao et al., 2024; Naghib et al., 2024).

**FIGURE 7:**
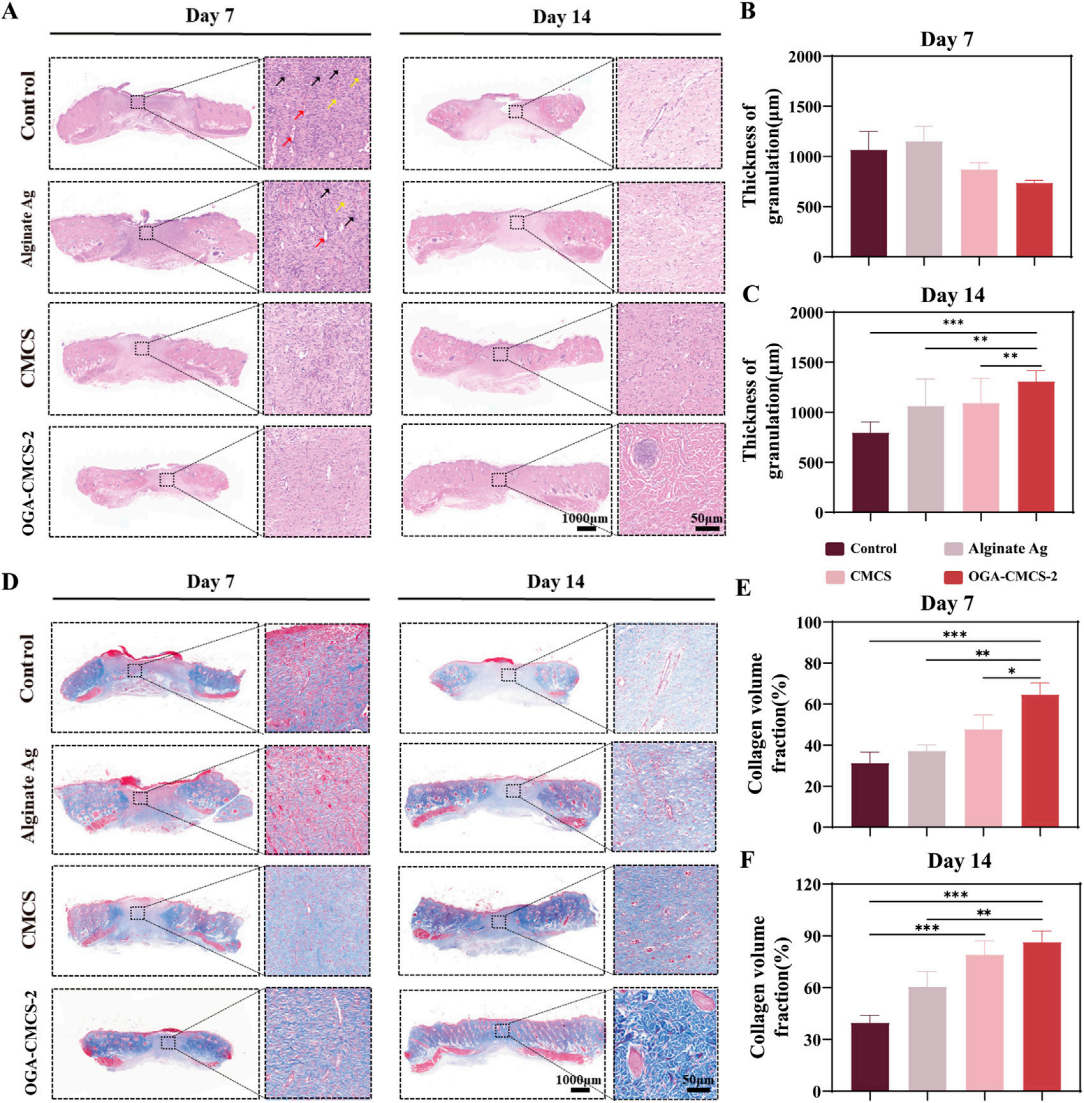
Efficacy of OGA-CMCS-2 hydrogel in rat model. **(A)** The HE-stained images of the treatment groups at days 7 and 14 (overall image scale bar = 1,000 μm; magnified local image scale bar = 50 μm). Red arrows indicate new blood vessels, black arrows indicate neutrophils, and yellow arrows indicate fibroblasts; **(B, C)** The thickness of newly formed granulation tissue in each treatment group at days 7 and 14 postoperatively. **(D)** Masson staining images of each treatment group at postoperative days 7 and 14 (scale bar for overall image = 1000 μm; scale bar for magnified local image = 50 μm); **(E, F)** Collagen volume fraction in each treatment group at postoperative days 7 and 14. (Note: n = 4, *P < 0.05, **P < 0.01, ***P < 0.001).

Figure 7A illustrates that, compared to the other three groups, the macroscopic degree of skin tissue structural abnormalities in the OGA-CMCS-2 hydrogel dressing group was markedly reduced by day 7, characterized by partial epidermal peeling, a reduction in inflammatory cell count, and a significant increase in the number of fibroblasts and neovascularization (Supplementary Figure S3). By day 14, the skin tissue structure in the OGA-CMCS-2 hydrogel dressing group had nearly returned to normal, exhibiting an intact epidermis, a further decrease in inflammatory cells, and an increase in the number of fibroblasts and new blood vessels, along with evidence of skin appendage regeneration. Skin appendages play a vital role in wound healing (Supplementary Figure S3). Hair follicles contain stem cells that migrate and differentiate to aid re-epithelialization and secrete growth factors that promote keratinocyte proliferation and migration, as well as neovascularization. Sebaceous glands produce sebum, which forms a protective barrier against water loss and pathogens and has anti-inflammatory properties. Sweat glands maintain microenvironmental stability by regulating temperature, humidity, and osmotic pressure at the wound site and secrete antimicrobial peptides to reduce the risk of bacterial infection, thus promoting wound healing (Liu et al., 2024; Zhou et al., 2024).

Figure 7B shows the quantitative analysis results of the neogranulation tissue thickness in each treatment group. On day 7, the neogranulation tissue thickness in the OGA-CMCS hydrogel dressing group was significantly lower than that in the blank control group and Alginate Ag group. On day 14, however, the neogranulation tissue thickness in the OGA-CMCS hydrogel dressing group was significantly higher than that in the blank control group and Alginate Ag group (Figure 7C).

Collagen is a major component of the extracellular matrix and is crucial for wound healing. Therefore, the assessment of collagen using Masson’s staining showed a significant increase in collagen deposition in the hydrogel group (Figures 7D–F). In conclusion, we concluded that the OGA-CMCS-2 hydrogel provids favorable conditions for wound healing, promoting granulation tissue regeneration and collagen deposition, and accelerating the development of new granulation tissue into normal tissue.

Granulation tissue serves to fill wound defects and forms the foundation for epithelial regeneration. It supplies essential nutrients and facilitates the removal of metabolic waste via the capillary network (Li et al., 2022). Fibroblasts are responsible for synthesizing the extracellular matrix, which is crucial for promoting tissue repair and remodeling. Inflammatory cells contribute to immune defense mechanisms. Throughout all phases of wound healing, granulation tissue assumes a pivotal role. A comprehensive understanding of its underlying mechanisms holds significant implications for advancing wound healing research and refining clinical treatment strategies (Zhou et al., 2024).

#### 3.12.3 The assessment of neovascularization in newly formed wound tissue

**FIGURE 8:**
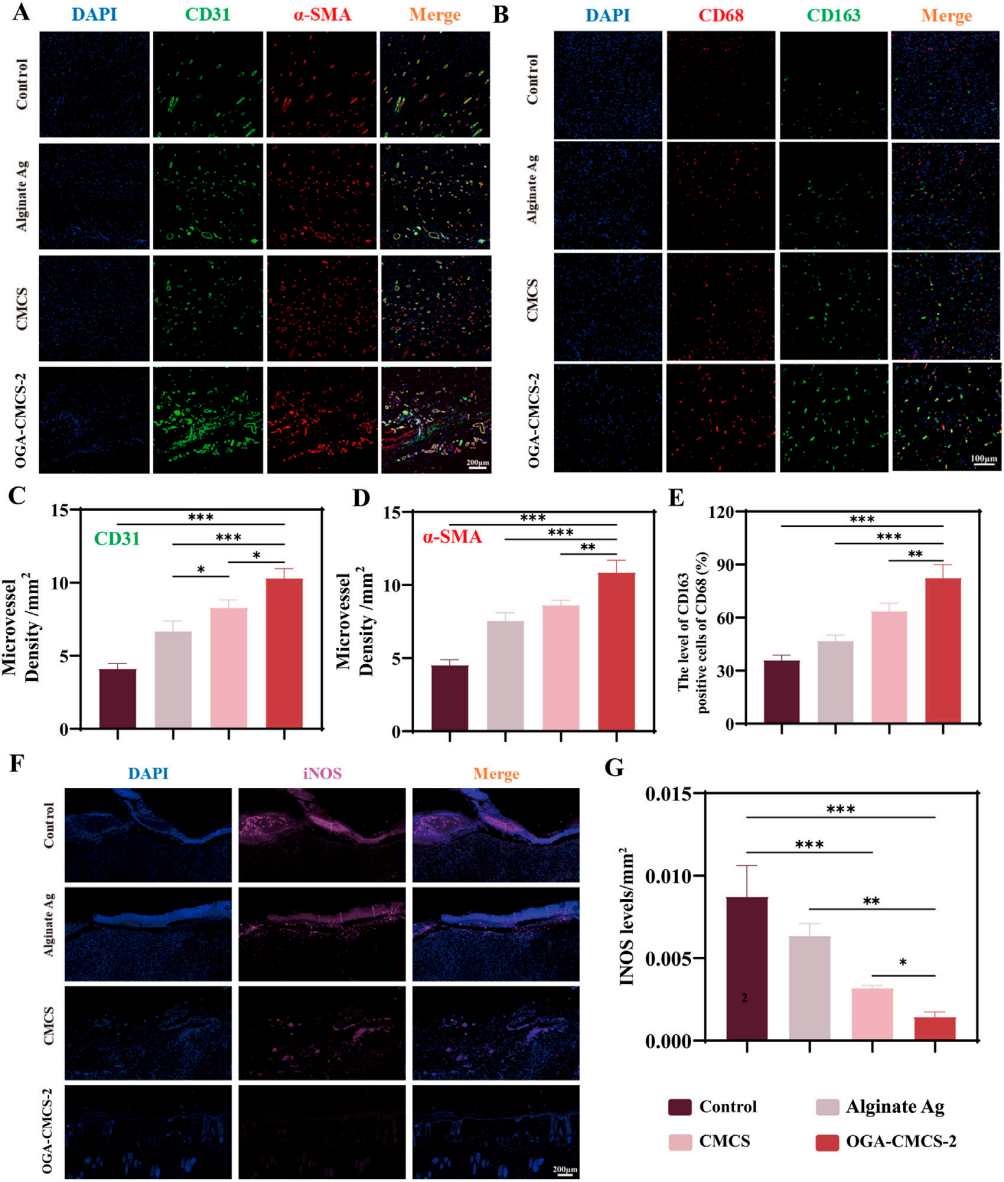
Histological and immunohistochemical analysis of hydrogels. **(A)** Representative images of microvessel immunofluorescence double staining of new tissue in the wound were captured on the 14th day after surgery. **(B)** Representative images of CD68 and CD163 immunofluorescence double staining in the new tissue of the wound were obtained from each treatment group on the 7th day post-surgery. **(C, D)** CD31 and α-SMA density analysis was conducted to evaluate facial neovascularization in each treatment group on day 14 after surgery. **(E)** The percentage of CD163/CD68-positive cells in the new tissue of the wound in each treatment group on the 7th day after surgery. **(F)** Representative images of newly formed tissue in each group on the 7th day after surgery were subjected to single-stain immunofluorescence for iNOS detection. **(G)** The average optical density of iNOS in the newly formed tissue in each treatment group on the 7th day after surgery was analyzed. (Note: n = 4, *P* < 0.05, **P < 0.01, ***P < 0.001).

The expression of CD31 indicates the presence of vascular endothelial cells, while α-SMA serves as a marker for α-smooth muscle actin. Immunofluorescence double staining these two markers was performed to assess microvascularization in new wound tissue (Figure 8A). Results from Figures 8C, D demonstrated that on the 14th day post-surgery, the OGA-CMCS-2 hydrogel dressing group exhibited a higher microvascular density compared to the other three groups. Further experiments (Supplementary Figure S4) confirmed that OGA-CMCS-2 possesses the capability to promote angiogenesis.

#### 3.12.4 The regulatory impact of hydrogel on macrophages in the newly formed wound tissue

iNOS is a marker of M1 macrophages, CD163 is a marker of M2 macrophages, and CD68 is a pan-macrophage marker. Therefore, the expression of these markers in the newly formed tissue on day 7 post-surgery can be evaluated by immunofluorescence to assess the regulation of OGA-CMCS hydrogel on macrophages in the newly formed tissue.

The results shown in Figures 8B, E indicate that the fluorescence intensity of M2 macrophages in the OGA-CMCS-2 hydrogel group is significantly enhanced compared to the other three groups. Meanwhile, Figures 8F, G show that the iNOS fluorescence intensity in the OGA-CMCS-2 hydrogel group is significantly lower than that of the other three groups. These results suggest that the OGA-CMCS-2 hydrogel can promote the transformation of macrophages from the M1 type to the M2 type. We employed flow cytometry analysis (Supplementary Figure S5) and real-time fluorescent quantitative polymerase chain reaction (q-PCR) for a more in-depth investigation (Supplementary Figures S6C, D). *In vitro* experiments demonstrated that the hydrogel was unable to directly convert macrophages from the M1 to the M2 phenotype (Supplementary Figure S5). It is postulated that during the process of wound healing, the hydrogel affects macrophage M1-M2 polarization by providing an appropriate wound environment (Supplementary Figures S6C, D) and effective antibacterial activity, thereby ultimately accelerating the healing process. Consequently, this process significantly mitigates the intensity of the inflammatory response and accelerates the wound healing and repair processes.

### 3.13 Expression of inflammatory factors within the wound

The persistence of the inflammatory response within the wound is a significant factor contributing to delayed healing, resulting in stagnation of the healing process. Consequently, on the seventh day post-surgery, the levels of key inflammatory factors (IL-6, TNF-α, IL-4, and IL-10) in the newly formed granulation tissue from the dorsal wounds of SD rats were quantified using ELISA (pg/mL).

**FIGURE 9:**
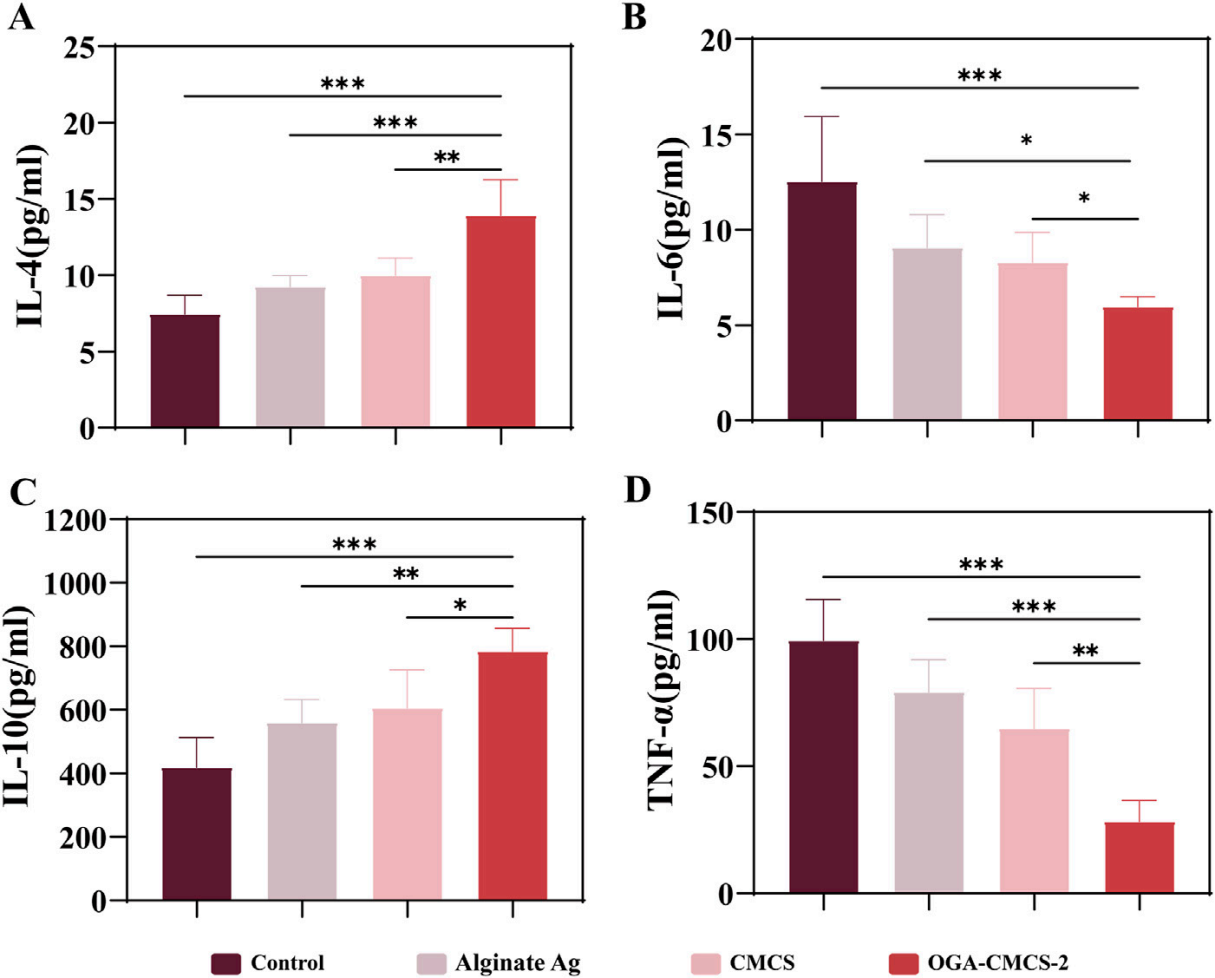
The outcomes from ELISA analyses conducted on the newly formed granulation tissue. **(A–D)** The ELISA results for IL-6, TNF-α, IL-4, and IL-10 in the newly formed granulation tissue.

As shown in Figure 9, the hydrogel exhibited significantly reduced expression levels of pro-inflammatory cytokines IL-6 and TNF-α, while the expression levels of anti-inflammatory cytokines IL-4 and IL-10 were markedly elevated compared to the control group. Additionally, notable statistical differences were observed between the hydrogel group and the other group. q-PCR experiments demonstrated that following hydrogel treatment, the expression levels of interleukin-10 (IL-10) were significantly upregulated, while those of tumor necrosis factor-α (TNF-α) were markedly downregulated (Supplementary Figures S6A, B). These findings robustly support the anti-inflammatory efficacy of OGA-CMCS and provide a critical molecular foundation for its application in inflammation intervention. These findings suggest that the OGA/CMCS hydrogel enhances the secretion of anti-inflammatory factors in newly formed granulation tissue while suppressing pro-inflammatory factor secretion, thereby effectively mitigating wound inflammation and accelerating granulation tissue regeneration, ultimately facilitating wound healing.

## 4 Conclusion

Through a comprehensive range of material characterization and *in vivo* and *in vitro* experiments, this study has demonstrated that the OGA-CMCS hydrogel dressing not only fulfills all clinical requirements (including rapid adhesion, gumming, viscoelasticity, swelling, injectability, and self-healing), but also exhibits excellent biocompatibility, antibacterial properties, rapid hemostatic performance, and the ability to promote accelerated repair of acute wounds. Notably, in wound healing models of animal studies, the incorporation of glycyrrhizic acid has been shown to effectively suppress inflammatory responses and promote the polarization of macrophages from the M1 phenotype to the M2 phenotype.

Compared to conventional wound dressings utilized in clinical settings, our hydrogel dressings exhibit substantial advantages across multiple dimensions. In terms of manufacturing, the production process is notably straightforward, eliminating the need for intricate and costly machinery and techniques, thereby significantly lowering the technical barriers and production costs. The entire process, from the acquisition of raw materials to the final product formation, can be executed in a highly efficient and cost-effective manner, rendering large-scale production both viable and economically advantageous.

Regarding its biological activity, our hydrogel exhibits numerous distinctive properties, including healing, injectability, anti-inflammatory effects, promotion of macrophage polarization, and antibacterial capabilities. Conventional wound dressings utilized in clinical settings often emphasize a limited range of functions (Gong et al., 2023). For instance, some traditional dressings are primarily designed for basic physical isolation and fluid absorption, yet they fail to actively facilitate the intricate physiological regulation required during the wound healing process (Wei et al., 2020).

In recent years, numerous researchers have dedicated their efforts to the development of various hydrogels for skin wound dressing applications. Among these, injectable hydrogels stand out as particularly promising candidates in the field of wound care due to their distinctive advantages in facilitating wound healing (Li et al., 2024). When utilized for skin wound repair, injectable hydrogels not only exhibit characteristics akin to conventional hydrogels, such as a three-dimensional network structure, superior water absorption and retention capabilities, and high porosity, but also fulfill several critical criteria (Li et al., 2023). Firstly, they can be administered in a localized or minimally invasive manner using fine needles, thereby eliminating the need for invasive surgical procedures (Zhao et al., 2023); Secondly, they possess the ability to conform to the desired shape within confined spaces, enabling them to fill irregularly shaped wounds (Lin et al., 2023). Thirdly, they demonstrate excellent biocompatibility, antibacterial properties, and minimal cytotoxicity (Li et al., 2024); Lastly, they exhibit effective hemostatic properties and can significantly enhance tissue regeneration (Chen et al., 2025).

Our hydrogels, owing to their injectable properties, can be readily applied to wounds of various shapes and depths. Upon application, they rapidly form a cohesive gel layer at the wound site, creating an optimal microenvironment for tissue repair. The antibacterial efficacy of the hydrogels efficiently resists external bacterial invasion, reducing the risk of wound infection, a critical aspect in clinical wound management. The anti-inflammatory properties of the hydrogels effectively mitigate the inflammatory response, minimizing the damage and irritation caused by inflammatory factors to surrounding healthy tissues, thereby accelerating the wound healing process. By promoting the polarization of macrophages, the hydrogels assist in regulating the body’s immune response, guiding the transformation of macrophages into phenotypes that facilitate tissue regeneration, thus further promoting the orderly progression of wound healing.

In summary, the synergistic effects of these biological activities render our hydrogel dressing superior in promoting wound healing, demonstrating significant advantages and application potential compared to conventional clinical wound dressings.

Unfortunately, there is currently no recognized hydrogel dressing available for the treatment of acute wounds in clinical practice. Therefore, this study can only be compared with existing clinical treatment methods. Additionally, it should be noted that this study solely demonstrates the cellular-level biocompatibility of hydrogels. Future studies using glycyrrhizic acid as the main biological material should include further safety tests to assess its histocompatibility, blood compatibility, and potential impact on various organs when used in dressings. Furthermore, considering glycyrrhizic acid’s role as an inhibitor of high-mobility group box 1 (HMGB1) target (Jeon et al., 2019; Mees et al., 2022), it would be worthwhile to investigate whether OGA-CMCS hydrogel dressing blocks pro-inflammatory positive feedback by inhibiting HMGB1 target in acute wounds to promote wound healing. Nevertheless, based on the experimental results obtained in this study, it is evident that this novel dressing containing glycyrrhizic acid as its primary component holds significant potential for repairing acute skin defect wounds.

## Data Availability

The original contributions presented in the study are included in the article/Supplementary Material, further inquiries can be directed to the corresponding authors.
