# Correction: An injectable, self-healing, anti-infective, and anti-inflammatory novel glycyrrhizic acid hydrogel for promoting acute wound healing and regeneration

**DOI:** 10.3389/fbioe.2025.1672232

**Published:** 2025-09-01

**Authors:** Qiyou Guo, Ruojing Li, Yeying Zhao, Huibo Wang, Wenqiang Luo, Junhao Zhang, Zhenlu Li, Peige Wang

**Affiliations:** ^1^ Department of Emergency Surgery, The Affiliated Hospital of Qingdao University, Qingdao, China; ^2^ Department of Emergency Medicine, Zhuji Affiliated Hospital of Wenzhou Medical University, Zhuji, Zhejiang, China

**Keywords:** glycyrrhizic acid, hydrogels, wound dressings, acute wounds, wound repair

There was a mistake in Figure 1E as published. After re-reviewing the manuscript carefully, we realized that an inadvertent error occurred in Figure 1, where the image of Figure 1E was an incorrect version. By referring the original experimental records of Electron microscope images of hydrogels, we have prepared a corrected version of the Figure 1E and the entire Figure 1 to rectify this mistake. This error does not affect the results and conclusions of this study. The corrected Figure 1 appears below.

**FIGURE 1:**
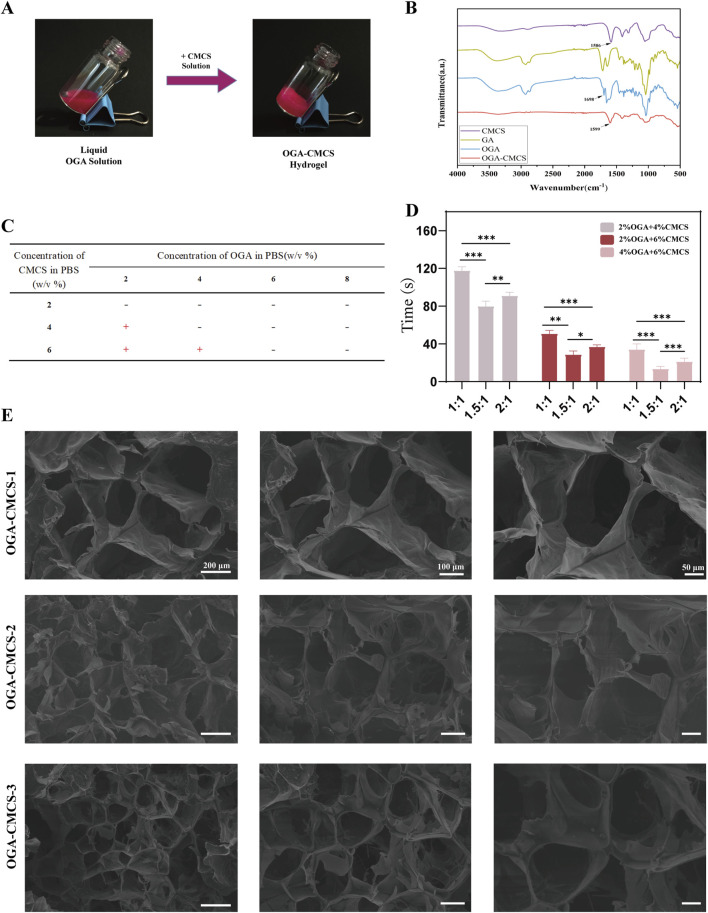
Synthesis of OGA-CMCS hydrogel. **(A)** Vial tilt test **(B)** Fourier transform infrared spectra of CMCS, OGA, GA, and OGA-CMCS hydrogels. **(C,D)** Gelation times of OGA-CMCS hydrogels with different mixing ratios. **(E)** Microstructures of OGA-CMCS-1, OGA-CMCS-2, and OGA-CMCS-3 hydrogels.

The original article has been updated.

