# Supplementary material for: An injectable, self-healing, anti-infective, and anti-inflammatory novel glycyrrhizic acid hydrogel for promoting acute wound healing and regeneration
Source: Front Bioeng Biotechnol. 2025 Jan 10;12:1525644. doi: 10.3389/fbioe.2024.1525644 (PMC11759265; doi:10.3389/fbioe.2024.1525644)
Supplement: Supplementary file 1 [file DataSheet1.docx]

***Supplementary materials***

**An injectable, self-healing, anti-infective, and anti-inflammatory novel glycyrrhizic acid hydrogel for promoting acute wound healing and regeneration**


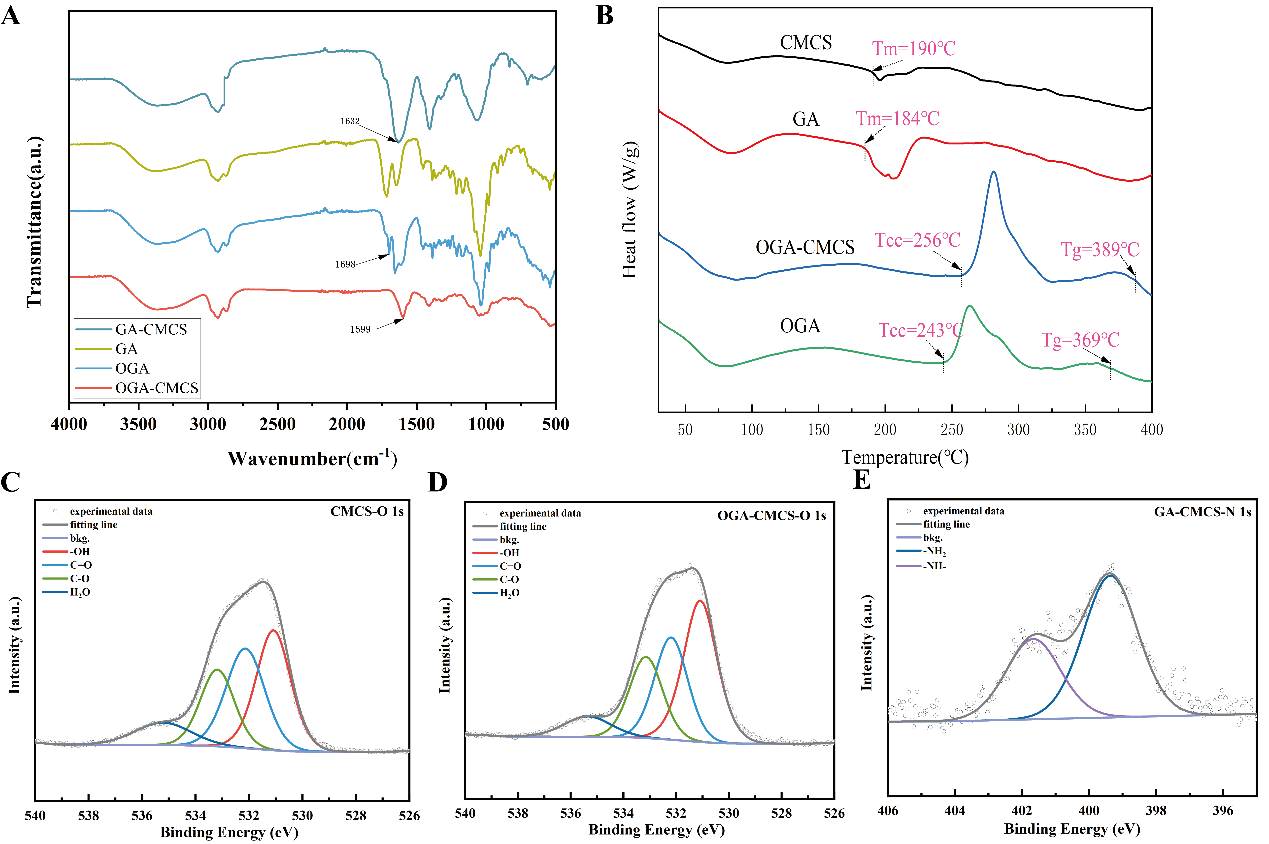


**Figure S1.** (A) Fourier transform infrared spectra of GA-CMCS, OGA, GA, and OPL-CMCS hydrogels. (B) DSC of the hydrogel. (C, D) The O 1s fine spectrum of CMCS and OGA-CMCS.

(E) The N 1s fine spectrum of GA-CMCS.


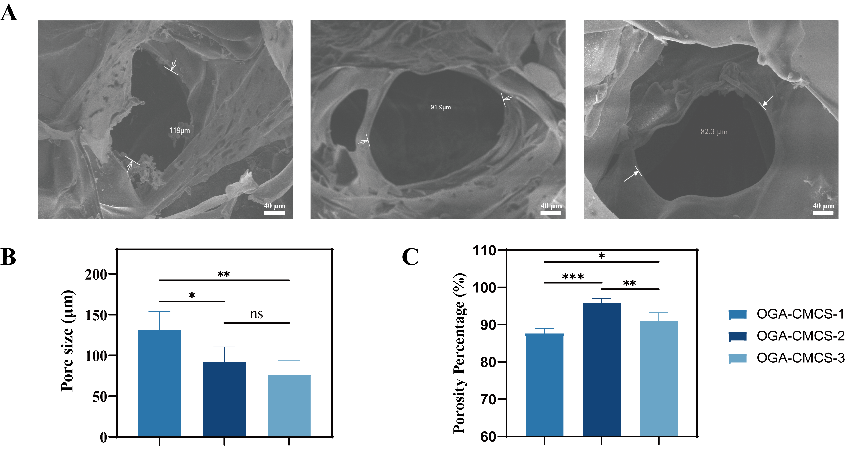


**Figure S2.** (A) Pore size diagram of hydrogel (B) Quantitative Analysis of Hydrogel Pore Size

(C) Porosity percentage of the hydrogels


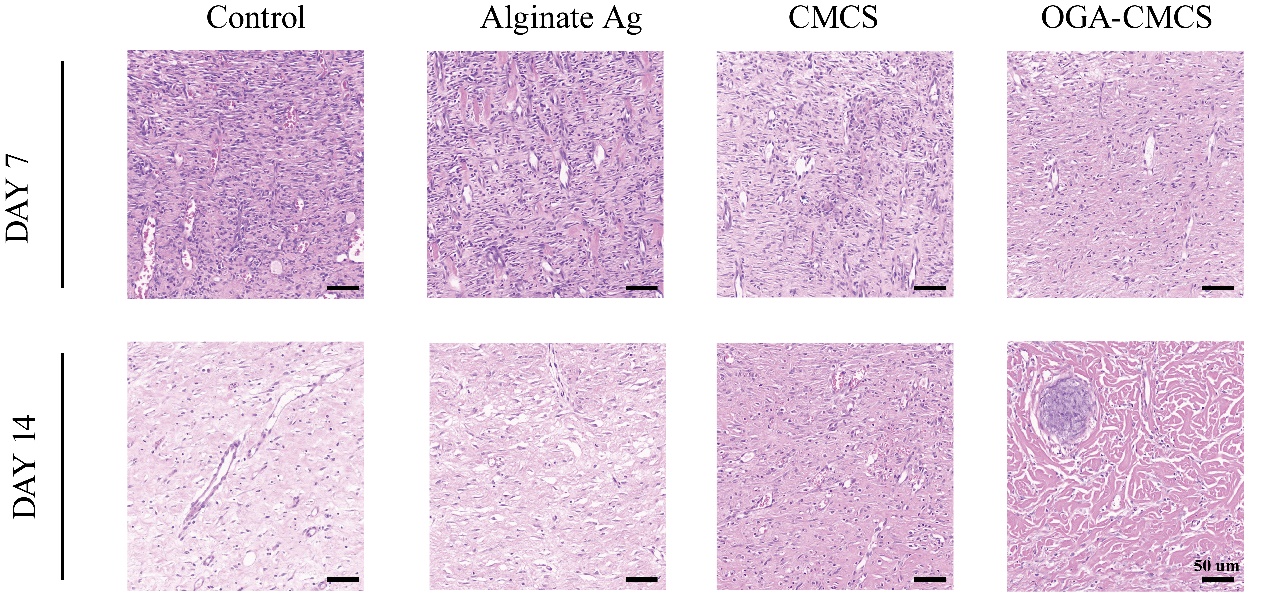


**Figure S3** Enlarged image of HE


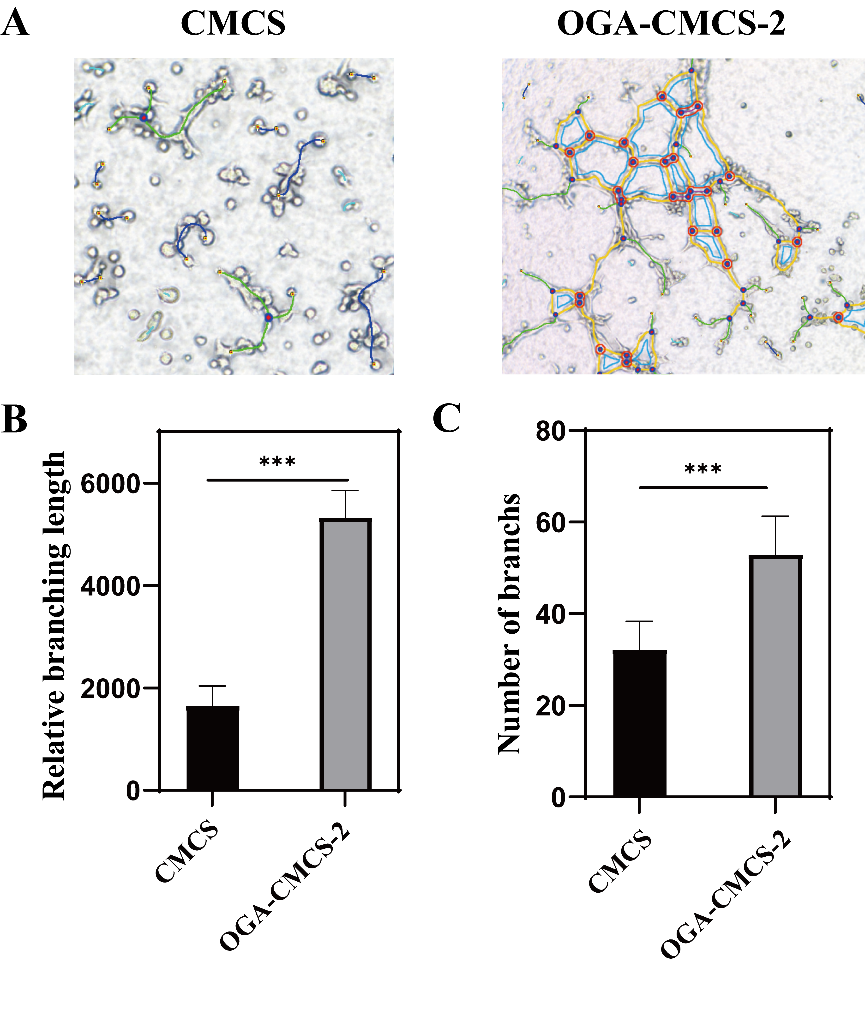


**Figure S4. Comparative Analysis of CMCS and OGA-CMCS-2 in Angiogenesis Assays.** (A) Images of tube formation by HUVECs after 6h of different treatments. (B、C) Quantitative analysis of branches and relative branching length.


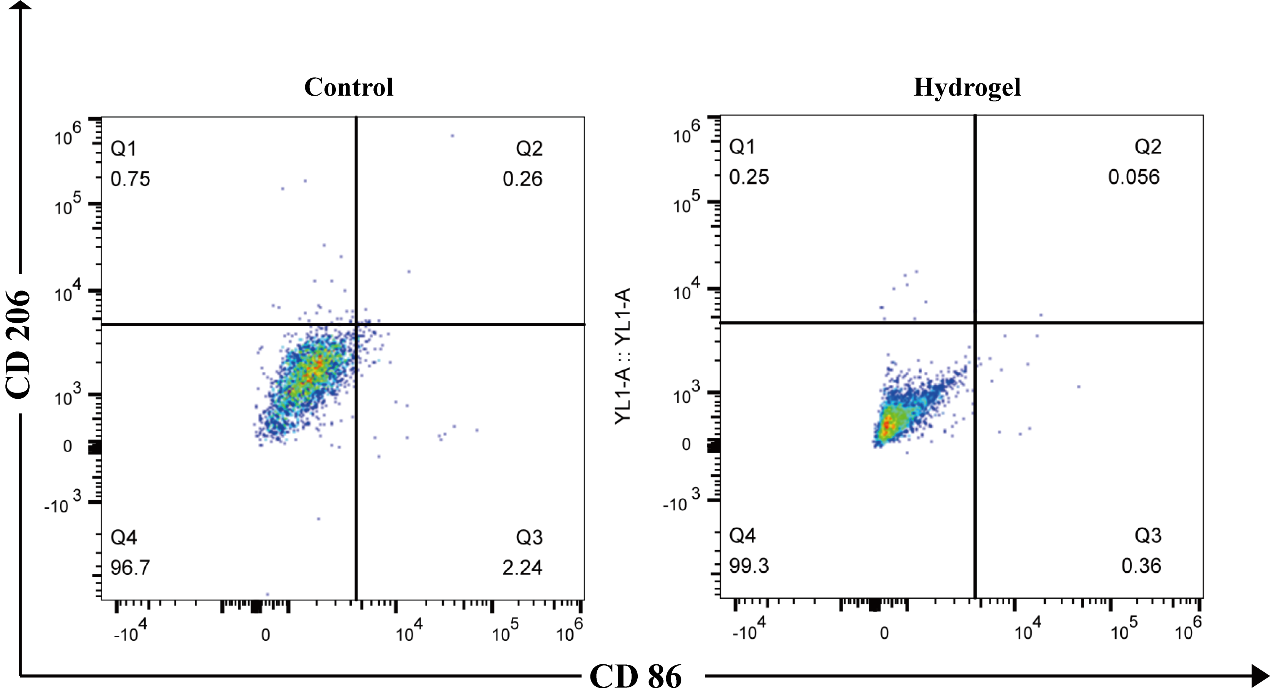


**Figure S5. Flow cytometry of M1&M2-specific markers**


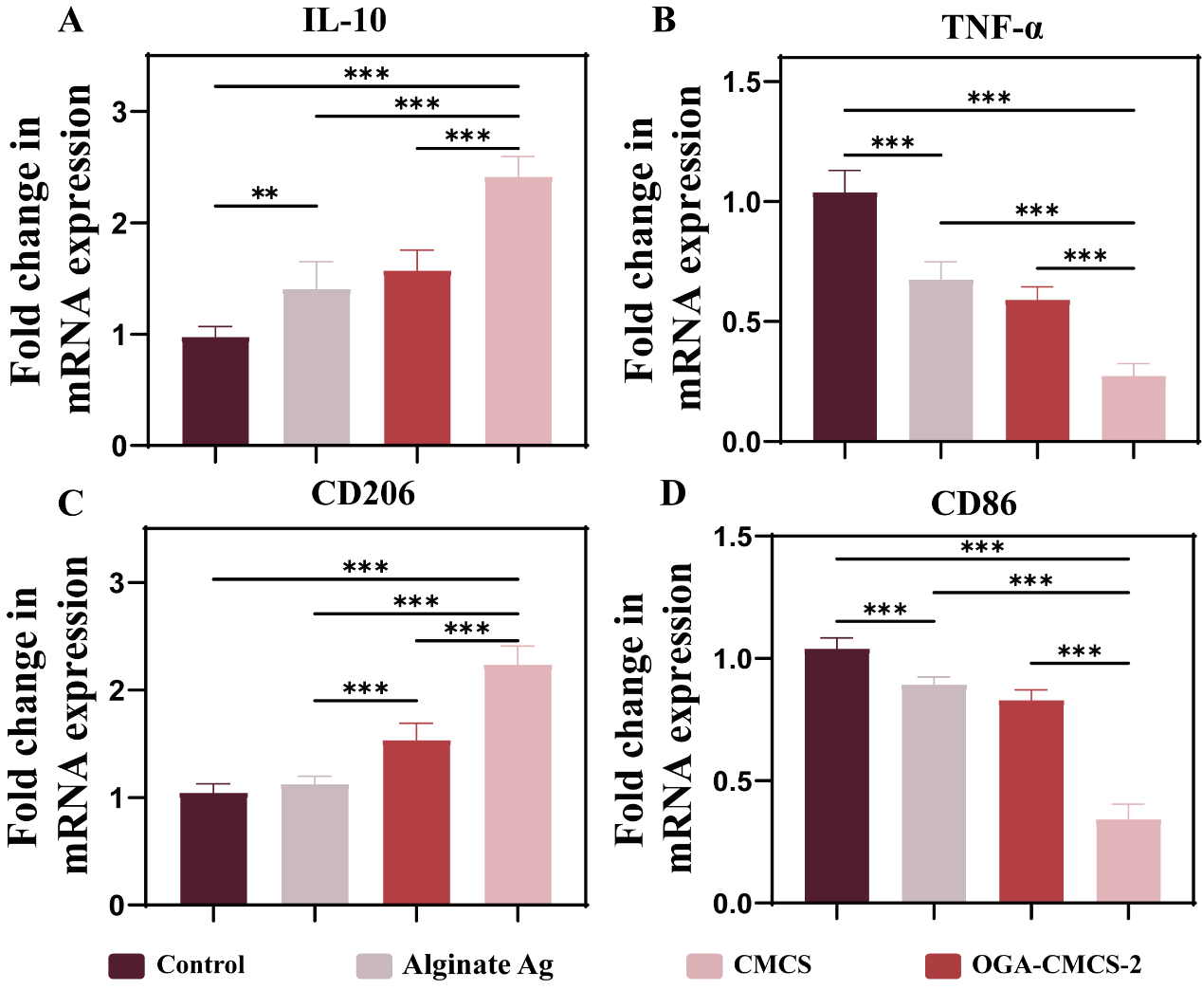


**Figure S6.** (A-D) Relative mRNA expression of IL-10, TNF-α, CD206, and CD86. (Note: n = 3, P < 0.05, ** P < 0.01, ***P < 0.001.)
